# Therapeutic Potential of Natural Compounds to Modulate WNT/β-Catenin Signaling in Cancer: Current State of Art and Challenges

**DOI:** 10.3390/ijms252312804

**Published:** 2024-11-28

**Authors:** Anna Gajos-Michniewicz, Malgorzata Czyz

**Affiliations:** Department of Molecular Biology of Cancer, Medical University of Lodz, 6/8 Mazowiecka Street, 92-215 Lodz, Poland

**Keywords:** cancer, WNT/β-catenin signaling, natural compounds, WNT modulators, clinical trials

## Abstract

Targeted therapies and immunotherapies have improved the clinical outcome of cancer patients; however, the efficacy of treatment remains frequently limited due to low predictability of response and development of drug resistance. Therefore, novel therapeutic strategies for various cancer types are needed. Current research emphasizes the potential therapeutic value of targeting WNT/β-catenin dependent signaling that is deregulated in various cancer types. Targeting the WNT/β-catenin signaling pathway with diverse synthetic and natural agents is the subject of a number of preclinical studies and clinical trials for cancer patients. The usage of nature-derived agents is attributed to their health benefits, reduced toxicity and side effects compared to synthetic agents. The review summarizes preclinical studies and ongoing clinical trials that aim to target components of the WNT/β-catenin pathway across a diverse spectrum of cancer types, highlighting their potential to improve cancer treatment.

## 1. Introduction

The WNT signaling pathway is evolutionarily conserved and holds a critical role in the development of organs and the maintenance of tissue homeostasis [1]. It has been demonstrated that progression, metastasis and resistance to treatment of various cancers can be attributed to the abnormal activation of WNT signaling; therefore, targeting the WNT/β-catenin pathway might be an effective treatment strategy [2,3,4].

Herbal preparations have been employed since ancient times as a fundamental source of therapeutic benefits for various diseases. Small molecules derived from natural products exhibit notable pharmacological activities, including antibacterial, anticancer, antioxidant and antifibrotic properties. Consequently, naturally sourced compounds are frequently utilized to treat diverse diseases, including cancers. Identifying bioactive compounds with significant anticancer activity across numerous plant species may facilitate the development of chemotherapy regimens incorporating these compounds alongside standard chemotherapeutic agents to enhance treatment efficacy and improve patient outcomes. During the last two decades, several natural compounds including curcumin, genistein, resveratrol, vitamin D, epigallocatechin-3-gallate (EGCG), apigenin, baicalin, galangin, silibinin, kaempferol, lycopene, naringenin, artemisinin, quercetin, fisetin, morin, aloe emodin, lupeol, alantolactone and tryptanthrin have been identified as potent modulators of the WNT/β-catenin signaling in various cancer types. Several of them are currently being tested in clinical trials. However, undesirable side effects and the development of resistance significantly restrict their efficacy in clinical applications [5]. There is mounting evidence that natural compounds can regulate the WNT/β-catenin signaling pathway [6]. Nevertheless, their clinical efficacy needs to be determined.

## 2. Brief Overview of WNT/β-Catenin Signaling Pathway

The WNT signaling pathway can be classified into two distinct pathways, canonical, defined by the intracellular accumulation of β-catenin, and non-canonical (WNT/Ca^2+^ pathway and planar cell polarity pathway), defined by β-catenin-independent actions [4].

The family of WNT ligands, crucial for stimulating WNT signaling consists of 19 cysteine-rich glycoproteins, each with a molecular weight of about 40 kDa and a length of approximately 350–400 amino acids, sharing a sequence identity of 20–85% [1]. Post-translational modifications, including glycosylation and palmitoylation, mediated by porcupine are essential for their biological activity [7]. Next to WNT ligands, the canonical WNT signaling pathway can also be potentiated by R-spondins (RSPOs), the cysteine-rich glycoproteins that belong to a superfamily of thrombospondin type 1 repeat-containing proteins. RSPO binds to both leucine-rich repeat-containing G-protein-coupled receptor 4/5/6 (LGR4/5/6) and ring finger protein 43/ zinc and ring finger 3 (RNF43/ZNF3) ligases, leading to the clearance of ZNRF3/RNF43 from the plasma membrane [8,9]. RNF43 and its homolog ZNRF3 promote the poly-ubiquitination of lysins in the cytoplasmic sequence of FZDs proteins, inducing endocytosis and the destruction of these receptors at the lysosome [10,11]. Therefore, LGR4/5/6-RSPO-RNF43/ZNF3 interaction results in increased levels of the FZDs on the plasma membrane, leading to increased WNT signaling [8]. Accordingly, in a non-activated state, in the absence of WNTs and RSPOs, ZNRF3 and RNF43 cause FZDs to be internalized and degraded [8].

The WNT/β-catenin signaling pathway is regulated by various antagonists. These antagonists include Dickkopf (DKK) proteins, secreted frizzled-related proteins (sFRPs), WNT inhibitory factor 1 (WIF1), WNT modulator in surface ectoderm (WISE), Kremen (KRM) and Cerberus protein (CER) [12,13,14]. Additionally, the activity of WNTs can be modulated by a highly conserved feedback antagonist, NOTUM, which functions as a deacetylase by removing a palmitate moiety from WNTs, thereby inducing their inactivation. WNT ligands bind to a transmembrane protein called Evenness interrupted/Wntless (EVI/WLS) and are transported through the Golgi apparatus to the plasma membrane [15]. The type of ligand present typically determines the mode of the WNT-signaling network. WNT1, WNT2, WNT3, WNT3A, WNT8a, WNT8b, WNT10a and WNT10b activate the canonical pathway, while WNT4, WNT5A, WNT5B, WNT6, WNT7a, WNT7b and WNT11 activate the non-canonical WNT-signaling pathway [13,16]. In the absence of WNTs, the receptors FZDs and LRP5/6 are located separately on the plasma membrane and the cytosolic β-catenin is captured by a “destruction complex” made up of adenomatous polyposis coli (APC), axis inhibition protein (AXIN), casein kinase 1 (CK1), glycogen synthase kinase 3β (GSK3β) and β-transducin repeat-containing protein (β-TrCP). Phosphorylated β-catenin is then continuously ubiquitinylated by β-TrCP, which leads to its degradation within the proteasome [4]. The activation of the WNT/β-catenin pathway is initiated by the binding of WNT to the FZDs and coreceptors, which are low-density lipoprotein (LDL)-receptor-related proteins 5 and 6 (LRP5 and LRP6). Ten types of FZDs have been identified in mammals (FZD 1–10), each belonging to the “Frizzled class” within the superfamily of G-protein coupled receptors (GPCRs). The N-terminus of FZDs contains a conserved cysteine-rich domain (CRD) with a hydrophobic cavity necessary for binding to the palmitoleate moiety of WNT ligands [17,18]. Upon the binding of the WNT ligand to the FZDs and LRP 5/6, disheveled protein (DVL) is recruited to the cell membrane to provide a platform for AXIN and GSK3β to bind and phosphorylate LRP5/6 (at five conserved PPPSP, also called the PPPSPXS) motifs, thereby preventing the constitutive degradation of β-catenin. As a result of this process, β-catenin becomes stabilized and moves to the nucleus, binds to transcription factors TCF/LEF (T-cell factor/Lymphoid enhancer factor) and recruits activators, such as CREB-binding protein (CBP)/p300, Pygopus (PYGO), B-cell lymphoma 9 (BCL9) and Brahma-related gene-1 (BRG1), leading to the expression of target genes [17,19]. The entry of β-catenin into the nucleus is a major signaling step in the canonical WNT pathway. β-catenin has no identifiable nuclear localization sequence (NLS) and exhibits the non-classical mode of nuclear import [20]. It has been found that the N-terminal, C-terminal tail and Armadillo repeats 10–12 of β-catenin can bind Nup358, Nup62, Nup98 and Nup153 of the nuclear pore complex (NPC), allowing β-catenin to enter into the nucleus through transient interactions [21]. In the nucleus, β-catenin acts as a regulator of gene expression for WNT target genes encoding various proteins that play crucial roles in cellular processes. This includes regulators of proliferation such as vascular endothelial growth factor (VEGF); fibroblast growth factor (FGF); c-JUN; and FOS-related antigen 1 (FRA1), regulators of the canonical WNT pathway such as WNT1-inducible signaling pathway protein 1 (WISP1), AXIN, DKK1, TCF, and LEF1, as well as matrix metalloproteinases and certain components of the extracellular matrix, cadherins, and lineage-specific proteins like microphthalmia-associated transcription factor (MITF) [22].

## 3. Preclinical and Clinical Studies Evaluating Synthetic Agents as Modulators of WNT/β-Catenin Signaling

In recent decades, novel targeted therapies and immunotherapies have provided great benefits for cancer patients. Nonetheless, the development of resistance limits the success and impedes the curative outcome [23,24]. Therefore, novel therapeutic targets are needed, and modulating WNT/β-catenin signaling is one of the strategies [25]. Aberrant WNT signaling has been implicated in various types of human cancers, contributing to malignant cell transformation, neoplastic proliferation, metastatic dissemination and resistance to treatment [26]. Moreover, the impact of abnormal canonical WNT signaling extends beyond cancer cells, as it significantly interacts with the microenvironment and the immune system dynamically [27]. Prolonged activation of the WNT/β-catenin pathway provides cancer cells with continuous self-renewal and growth and is associated with resistance to therapy [28]. Genetic and epigenetic alterations that affect constituents of WNT/β-catenin signaling pathways are tissue-specific and differ between cancers. Understanding at which level these mutations occur within the pathway is critical to developing new therapeutic strategies [29]. In addition to the common mutations of APC in colorectal cancer and CTNNB1 in hepatocellular carcinoma, dysregulation of various extracellular modulators of WNT signaling (e.g., DKKs, sFRPs, and WIF1) and WNT receptor abundance also plays a role in the development of cancer [28]. Moreover, high expression of vacuolar H^+^-ATPase (v-ATPase), an electrogenic H^+^ transporter required for canonical WNT-signaling activation, that triggers abnormal WNT/β-catenin-signaling is observed in prostate, breast, colorectal, pancreatic and ovarian cancer cells [30]. However, the canonical WNT signaling is highly complex and context-dependent in various cancers and high levels of nuclear β-catenin do not always indicate poor prognosis. Therefore, it is necessary to consider the cell type-specific background to understand the cellular outcome of aberrations in WNT-signaling [31]. While detected in benign nevi, nuclear β-catenin is downregulated during melanoma progression [31,32,33]. This contrasts with other cancers, e.g., colorectal cancer [34], hepatocellular carcinoma [35] pancreatic cancer [36], lung cancer [37] or ovarian cancer [38], in which nuclear β-catenin is a driving force of both initiation and progression. Moreover, alterations in WNT/β-catenin signaling have been associated with phenotype switching of melanoma cells, leading to a transition from a highly proliferative/non-invasive state to a slow proliferative/metastatic condition [39]. It has been observed that β-catenin-suppressed invasion occurs through a cell-type specific mechanism involving MITF [31]. On the other hand, activation of β-catenin is crucial for bypassing melanocyte senescence, ultimately leading to melanocyte transformation [40,41], and low efficacy of immunotherapy is observed in melanomas with elevated levels of β-catenin. It has been found that dysregulation of WNT/β-catenin signaling is strongly associated with the biological function of immune cells and immune evasion that play a central role in non-responders or resistant patients receiving immune checkpoint inhibitors (ICIs) [42]. Therefore, the efficacy of ICIs treatment could be significantly improved through combined therapy with molecules targeting the canonical WNT signaling pathway [43]. Furthermore, there is a crosstalk between WNT signaling pathways and other signaling pathways crucial for melanoma development such as MAPK/ERK and phosphoinositol-3-kinase (PI3K)/protein kinase B (AKT) signaling. Therefore, considering the role of the WNT/β-catenin pathway in developmental and adult stages and its role in melanoma, the effective and beneficial modulation of the canonical WNT pathway is a challenging task [19]. While targeting the WNT/β-catenin signaling pathway is the subject of a number of clinical trials evaluating drug efficacy in various cancer types (Table 1), studies investigating modulators of WNT/β-catenin pathway in melanoma are still in the preclinical stage with only a limited number of clinical trials. Similarly, in the context of hepatocellular carcinoma, preclinical studies have demonstrated the potential efficacy of small molecules, monoclonal antibodies and plant-derived agents in the regulation of WNT/β-catenin signaling. However, the progression of these therapeutic agents to clinical trial stages remains limited. The clinical advancement of canonical WNT pathway inhibitors for patients with hepatocellular carcinoma faces substantial challenges due to the critical role of WNT/β-catenin signaling in liver homeostasis and regeneration, and its interaction with other signaling pathways [35]. Recent studies have revealed that the activation of PTEN-induced AKT signaling, alongside the suppression of the WNT/β-catenin pathway, is essential for modulating the ratio of LGR5+ cells in liver cancer, suggesting that dual modulation may represent a promising novel approach for the treatment of liver cancer. Additionally, LGR5 has been identified as a significant marker for cancer stem cells (CSCs), highlighting its relevance in cancer research and its prospective applications in therapeutic strategies [44,45].

The canonical WNT pathway can be modulated at various stages, and blocking the production of active WNTs with drugs is one of the strategies. This can be achieved by targeting a key enzyme in WNT biosynthesis, the membrane-bound O-acyltransferase, porcupine. C59 [91] and WNT974 (also known as LGK974) [45,46] are porcupine inhibitors being under investigation in several cancer types, including melanoma. C59, the commercial name of 2-(4-(2-methylpyridin-4-yl)phenyl)-*N*-(4-(pyridin-3-yl)phenyl) acetamide, synergizes with the anti-CLA-4 antibody in the B16 melanoma model, indicating a possible enhancement in antitumor immunity through a synergistic mechanism [91]. LGK974 is under clinical investigation in melanoma, pancreatic cancer, triple-negative breast cancer, cervical squamous cell cancer, esophageal squamous cell cancer, lung squamous cell cancer (NCT01351103) and BRAF mutant colorectal cancer (NCT01351103 and NCT02278133). Another way of targeting WNT/β-catenin signaling is by affecting the WNT ligands with antibodies, such as WNT-2Ab, a monoclonal antibody with the potential to induce cell death in melanoma cells [92]. WNT-signaling inhibitors can also target FZDs-DVL interaction, e.g., FJ9, which has been found to downregulate WNT/β-catenin signaling and induce apoptosis in lung cancer and melanoma cells [93]. Tankyrases (TNKS and TNKS2) are enzymes that PARylate AXIN1 and AXIN2, resulting in the subsequent ubiquitination and proteasomal degradation of AXIN1/2 [94,95,96]. Tankyrase inhibitors XAV939 [97] and G007-LK [98] represent a promising approach in melanoma therapy. It has been found that G007-LK, can overcome WNT/β-catenin-mediated resistance to immune checkpoint inhibitors [98], whereas nanoparticle formulation for XAV939 (XAV-Np) is efficacious in inhibiting melanoma cell viability, migration and tumor progression in a mouse model of conjunctival melanoma [97]. Blocking the interaction between β-catenin and TCF4 can also act as a novel anticancer strategy [99]. It has been observed that PKF 115–84, a small-molecule inhibitor of β-catenin/TCF/LEF complex downregulated β-catenin expression and β-catenin transcriptional activity, resulting in a dose-dependent reduction in viability of melanoma cells [100]. Moreover, Sinnberg et al. [101] have reported that PKF115–584 decreased melanoma cell migration in vitro and blocked the neural crest migration of melanoma cells in a chick embryo in vivo [101]. Pentoxifylline, a drug approved by the FDA for the treatment of peripheral arterial disease has also been found to inhibit WNT/β-catenin signaling, as it effectively reduced the level of active β-catenin in the nucleus of patient-derived melanoma cells with a high basal expression of β-catenin [102]. Recent studies have also shown that disrupting the interaction between β-catenin and BCL9 can also inhibit oncogenic WNT/β-catenin activity [85,86] and ST316, a novel peptide antagonist of β-catenin that inhibits the interaction with BCL9 and has recently entered the path of clinical trials (NCT05848739) and its activity is investigated in several cancer types including melanoma.

Pharmacological activation of the WNT pathway has also been considered as a potential therapy for melanoma, as riluzole treatment increased WNT/β-catenin signaling to enhance the pigmentation, reduce the proliferation of melanoma cells, and decrease lymph node metastasis in vivo in a mouse melanoma model [103]. Riluzole, an inhibitor of metabotropic glutamate receptor 1 (GRM1) signaling and an FDA-approved drug for amyotrophic lateral sclerosis (ALS) treatment, has been used in an already completed clinical trial (NCT00866840) evaluating riluzole in treating patients with stage III/IV melanoma [90]; its use combined with sorafenib is evaluated in phase I of a clinical trial in patients with advanced solid tumors (NCT01303341). LY2090314, a selective small-molecule inhibitor of GSK3α/β, stabilized β-catenin and stimulated the expression of AXIN2 in A375 melanoma cells, causing a tumor growth delay in vivo both as a single agent and in combination with dacarbazine (DTIC), and induced apoptotic cell death in melanoma cell lines irrespective of the BRAF mutation status [104].

Unfortunately, agents targeting WNT/β-catenin signaling frequently exhibit severe side effects that impair tissue homeostasis and regeneration, and the off-target effects of WNT inhibitors remain an unresolved issue. Natural compounds could be important not only for developing less toxic and more efficient therapeutic strategies when combined with standard anticancer drugs but also for potentiating the efficacy of ICI treatment. This novel treatment approach investigating the therapeutic potential of a combination of vitamin D (as a co-drug) and curcumin (as a supplement) alongside an immunomodulatory cocktail that includes the anti-PD-L1 pembrolizumab in patients with cervical cancer, endometrial carcinoma, or uterine sarcoma has already entered the path of clinical trials (NCT03192059, phase II/completed) [105,106]. It has been revealed some patients derived benefits from the therapy; however, the preclinical effectiveness of the combination of pembrolizumab with a drug cocktail enriched with curcumin followed by radiotherapy did not meet the expectations of clinical activity [105].

## 4. Characterization of Natural Compounds Modulating the WNT/β-Catenin Pathway in Cancer

**Figure 1:**
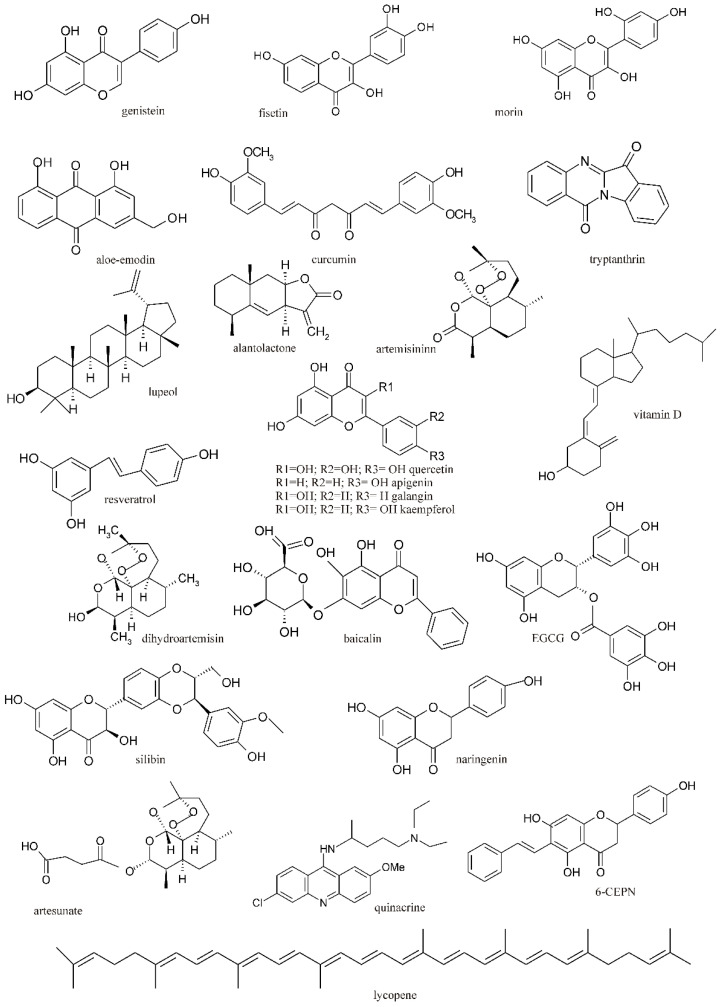
The chemical structures of natural compounds targeting WNT/β-catenin signaling in various cancers.

Natural products have been employed in traditional medicine across different cultures around the world for a long time. Recently, there has been a resurgence of interest in natural products as alternatives to synthetic medications or complementary therapies to conventional treatments. The historical use and continued investigation of natural products highlight their potential as a valuable tool in the fight against cancer [5]. As the deregulated WNT/β-catenin signaling pathway has been implicated in various cancer types, the identification of natural modulators of this pathway has already attracted considerable attention from the scientific community [43]. Natural compounds have been found to modulate the WNT/β-catenin pathway through different molecular mechanisms, including (1) GSK-3β activation, (2) the inhibition of β-catenin nuclear translocation, (3) the inhibition of β-catenin /TCF interaction, (4) promoting β-catenin protein degradation, (5) the induction of E-cadherin expression, (6) the induction of DKK1 expression, (7) the destabilization of the WNT-FZDs-LRP complex, and others. Moreover, the natural compounds have multiple targets involved in canonical WNT signaling; therefore, the mode of their action depends on the cell type and the state of dysregulation of the WNT/β-catenin pathway in specific diseases [43]. The majority of these therapeutic candidates are still in the preclinical development stage; however, some natural compounds have already entered the path of clinical trials. Preclinical and clinical therapeutic interventions with different natural compounds targeting the WNT/β-catenin signaling pathway in various cancer models are summarized in Table 2 and illustrated in Figure 1.

In the context of the regulation of the canonical WNT signaling pathway in cancer, a variety of natural compounds that belong to flavonoids, anthraquinones, terpenoids, alkaloids and curcuminoids are investigated in preclinical and clinical studies. The following section will focus on the ability of these compounds to modulate the WNT/β-catenin signaling in melanoma and other cancer types.

### 4.1. Phenolics

#### 4.1.1. Flavonoids

##### Quercetin

Quercetin [2-(3,4-dihydroxyphenyl)-3,5,7-trihydroxy-4H-chromen-4-one] is a natural flavonoid compound that is found abundantly in onions and other dietary sources, including mangoes, grapes, cherries, apples, buckwheat, plums, tomatoes and tea. It possesses multiple pharmacological activities including antioxidant, antiaging, antiviral (including anti-SARS-CoV-2) and anti-inflammatory properties [164,165]. The activity of quercetin has already been evaluated in various cancers, including lung cancer [166], prostate cancer [167], liver cancer [168], breast cancer [169], colon cancer [170], cervical cancer [171], thyroid cancer [151] and melanoma [110]. Numerous studies have shown that its anticancer properties are mediated through various signaling pathways, e.g., p53, nuclear factor-kappa B (NF-κB), MAPK, JAK/STAT, PI3K/AKT and WNT/β-catenin. Apart from regulating these pathways, quercetin also controls the activity of oncogenic and tumor suppressor non-coding RNAs (ncRNAs) [172]. It has been found that quercetin induced apoptosis in melanoma cells and destabilized the WNT-FZD-LRP6 complex by suppressing DVL2 and AXIN2, which resulted in decreased β-catenin and the inhibition of the WNT-responsive gene encoding cyclin D1 (CCND1) and cyclooxygenase (COX2). Moreover, co-treatment with curcumin-induced apoptosis by the downregulation of BCL2 and the induction of caspase 3/7 [110]. The modulation of WNT/β-catenin signaling has also been observed in other cancer types, as quercetin has inhibited the binding between β-catenin TCF [149] and inhibited GSK-3β phosphorylation [150] in colorectal cancer and induced significant anticancer effects against triple-negative breast cancer (TNBC) [171] and thyroid cancer [151] by the increased E-cadherin expression [151,173].

Although quercetin has many health benefits, its oral bioavailability is relatively poor and affected by various factors that limit its application as a clinically therapeutic agent. The water-insoluble nature of quercetin is the most significant reason why it cannot be absorbed into the intestinal tract. Although it can quickly penetrate the phospholipid bilayer of Caco-2 cells due to its hydrophobic properties, it cannot pass through the mucus layer surrounding the gastrointestinal tract, which contains 90% water content. Therefore, various approaches to increase its hydrophilicity, e.g., cyclodextrin inclusion, liposomes, micelles, and nanosuspensions, are investigated [174].

##### Fisetin

Fisetin (3,3′,4′,7-tetrahydroxyflavone) is a flavonoid abundantly present in a variety of fruits and vegetables, including, apples, strawberries, grapes, persimmon, mangoes, cucumbers, tomatoes, onions, nuts and wine [175,176]. Fisetin offers various pharmacological benefits, including antioxidant, anti-inflammatory, antiangiogenic and anticancer activity [176,177]. Fisetin has been shown to exert anticancer activities, including the inhibition of cell proliferation, angiogenesis, migration, oxidative stress and the induction of apoptosis [177]. It has been found that fisetin exhibits potency against multiple cancer types, including lung [178,179,180], breast [181], prostate [182], colon [183], bladder [184], renal [185], bone [186], pancreatic cancer [187,188], liver [189], oral [190], stomach [191], blood [192], ovarian [193], cervical [194] and melanoma [152,175,176]. These results suggest that fisetin might be a promising therapeutic candidate for cancer treatment. Anticancer properties of fisetin have been ascribed to its involvement in a plethora of signaling pathways, including VEGF, MAPK, NF-κB, PI3K/Akt/mTOR and Nrf2/HO-1, WNT/β-catenin and ERK signaling [176,177]. Syed et al. [152] highlighted the potential of fisetin as a therapeutic option for melanoma treatment via the modulation of the WNT/β-catenin signaling pathway, as the increase in endogenous WNT inhibitors DKK1 and WIF was concomitant with the decrease in the expression of coreceptors FZDs/LRP-6 and DVL has been observed in fisetin-treated melanoma cells. Moreover, the phosphorylation of GSK-3β has been found to be reduced by fisetin, accompanied by a decrease in the stability of β-catenin due to the presence of β-TrCP. As a result, the significant reduction in the protein levels of β-catenin/TCF targets, including c-Myc, BRN2 and MITF has been observed, including in vivo experiments conducted on tumor xenografted mice [152]. Malagoda et al. [195] have reported that high concentrations of fisetin (≥50 µM) decreased the viability of B16F10 melanoma cells, whereas fisetin at lower concentrations (≤20 μM) significantly increased melanin content in mouse melanoma cells and in vivo in a zebrafish larvae model. Based on the results of molecular docking studies, it has been found that fisetin binds to the non-ATP-competitive site of GSK-3β. This leads to the activation of β-catenin signaling, which in turn increases the expression of MITF and tyrosinase in both mRNA and protein levels. This suggests that fisetin promotes melanogenesis by inhibiting GSK-3β and releasing β-catenin [195]. It has also been revealed that fisetin downregulated β-catenin, TCF4 β-catenin target genes, cyclin D1 and matrix metalloproteinase 7 in colon cancer cells [153].

However, administering fisetin can be challenging due to its low water solubility, which limits its biological effects. Therefore, nanoemulsion formulations have been explored to address the issue of delivering fisetin to cancer cells more effectively. Nanoemulsion offers small particle sizes, high drug solubility and loading, good stability, sustained drug release and low toxicity. Another promising way to improve the effectiveness of fisetin is liposome encapsulation, which increases its delivery and absorption [177].

##### Morin

Morin (3,5,7,2′,4′-pentahydroxyflavone) is a polyphenol compound originally isolated from *Moraceae* family plants. It can be found in various fruits and vegetables, including mulberries, figs, almonds, onions, osage oranges, tea, coffee, guava leaves, old fustic and apples [196]. In the last decade, this dietary flavanol has received a lot of attention due to its promising pharmacological activities and therapeutic potential. It has been found that morin possesses various beneficial properties such as anti-inflammatory, antioxidant, neuroprotective, antihyperlipidemic, antiviral, antiallergic and anticancer effects [154,196]. Morin has been shown to suppress the proliferation and induce apoptosis in tumor cells, e.g., breast cancer [197], ovarian cancer [198], colorectal cancer [199], hepatocellular carcinoma [200], lung [201], leukemia [202] and melanoma [154,203]. It has been revealed that a morin-induced overexpression of miR-216a in CD133^+^ melanoma subpopulation inhibited WNT3A expression through miR-216a, which directly targets WNT3A 3′-UTR. Morin treatment significantly reduced the viability of CD133^+^ melanoma cells and decreased the expression of stemness markers such as CD20, CD133 and CD44. Moreover, morin treatment significantly reduced tumor size in a melanoma xenograft model [154]. Moreover, Lee et al. [203] have reported that morin inhibited melanoma cell growth and promoted apoptosis by downregulating antiapoptotic MCL-1 and BCL-2. This mechanism is partly regulated by the ROS-linked suppression of specificity protein 1 (SP1) expression [203]. However, low aqueous solubility and decreased intestinal absorption significantly limit the therapeutic application of morin. Therefore, various techniques are examined to overcome the low oral bioavailability of morin. Choi et al. [204] proposed a morin-loaded mixed micelle formulation consisting of morin-PluronicF127-Tween80 that significantly increased the bioavailability of morin [204].

#### 4.1.2. Anthraquinones

##### Aloe Emodin

Aloe emodin (1,8-dihydroxy-3-(hydroxymethyl)-anthraquinone) is a naturally occurring anthraquinone derivative and active ingredient of Chinese herbs, such as *Aloe vera*, *Rheum palmatum* L., *Cassia occidentalis* and *Polygonum multiflorum* Thunb. Aloe emodin has various pharmacological benefits, including anti-inflammatory, antiviral, antibacterial, hepatoprotective and neuroprotective activity [205]. Moreover, it is a promising anticancer agent that has been investigated in various tumors, including bladder cancer [206], cervical cancer [207], colon cancer [208], gastric cancer [209], melanoma [210], lung cancer [211], liver cancer [212], nasopharyngeal carcinoma [213], oral cancer [214], ovarian cancer [215], prostate cancer [216] and tongue cancer [217]. It has been found that aloe emodin inhibited the viability of A375 and SK-MEL-28 melanoma cells in a dose-dependent manner. Moreover, aloe emodin treatment reduced the migrative and invasive properties of melanoma cells and significantly inhibited the growth of A375 and SK-MEL-28 cells in vivo in a nude mouse transplanted tumor model. Mechanistically, it has been revealed that aloe emodin inhibited the expression levels of WNT3a and p-GSK3-β and promoted β-catenin phosphorylation, leading to the further degradation of β-catenin [155]. Another study has also demonstrated significant antineoplastic and immunomodulatory properties of aloe emodin against M14, SK-MEL-110 and SK-MEL-28 human melanoma cell lines, which was achieved by upregulating the expression of inflammation-associated factors such as interleukin (IL)-2, IL-12, GM-CSF and interferon (IFN)-γ [210]. The antiproliferative efficacy of aloe emodin has also been revealed in androgen-independent DU145 prostate cancer cells, where an aloe emodin treatment resulted in reduced WNT2 and β-catenin mRNA together with decreased β-catenin with their target genes, including cyclin D1 and c-myc [156].

However, the clinical application of aloe emodin is limited due to its hydrophobic character, poor intestinal absorption, short elimination half-life and low bioavailability in vivo [205]. To enhance the water solubility and oral bioavailability of aloe emodin, it was loaded into micelles that markedly enhanced the oral bioavailability of aloe emodin [218].

#### 4.1.3. Curcuminoids

##### Curcumin

Curcumin (1,7-bis-(4-hydroxy-3-methoxyphenyl)-hepta-1,6-diene-3,5-dione) is a major active component of the spice turmeric (*Curcuma longa*), a plant related to the ginger family (*Zingiberaceae*). Curcumin exhibits antibiotic, anti-inflammatory, anti-aging and anticancer activity as suggested by in vitro and in vivo studies and clinical trials [219,220,221,222]. The anticancer activity of curcumin was widely investigated and reported in, e.g., colorectal cancer [110,223], breast cancer [224,225], chronic myeloid leukemia [226], pancreatic cancer [227], glioblastoma [228], ovarian cancer [229], head and neck squamous cell carcinoma [230], bladder cancer [231] and erythroleukemia [232]. The antiproliferative effects observed in vitro and in vivo in various cancer models have prompted the investigation of curcumin’s pharmacological potential in clinical trials. In the past decade, 47 clinical studies have evaluated curcumin’s potential as an antitumor agent, and only a few of them have been completed with results (https://clinicaltrials.gov, 12 July 2024 ). Even though a potential anticancer effect has been demonstrated in patients, further studies are needed to confirm the use of curcumin as a preventive and therapeutic treatment, either on its own or in combination with standard therapies. Several lines of evidence have suggested that the biological effect of curcumin is mediated through the modulation of diverse signaling pathways, including the WNT/β-catenin pathway, various transcription factors, and miRNAs. It has been found that curcumin targets different critical components of the WNT/β-catenin pathway involved in the initiation, progression and drug resistance of different cancers [43]. Srivastava et al. [110] have found that a concentration-dependent reduction in DVL2 and AXIN2 by curcumin resulted in the destabilization of the WNT-FZD-LRP6 complex. In consequence, the levels of β-catenin and β-catenin target genes, CCND1 and COX2 have been decreased. It has also been revealed that curcumin showed antiproliferative and proapoptotic activity in melanoma cells via the BCL-2-mediated pathway. Furthermore, curcumin and quercetin co-treatment resulted in the enhanced inhibition of the proliferation of melanoma cells [110]. Moreover, curcumin repressed the proliferation, migration, and invasion of melanoma cells via enhancing miR-222-3p, which targeted SOX10 [233]. Apart from melanoma, curcumin effectively modulates the WNT/β-catenin pathway in breast cancer, as it inhibited proliferation and induced the apoptosis of breast cancer cells through the inhibition of DVL, β-catenin and cyclin D1 [111], as well as through the downregulation of phospho-GSK3β and β-catenin [112]. Similarly, curcumin treatment inhibited the proliferation of non-small-cell lung cancer (NSCLC) and medulloblastoma cell lines, through the inhibition of phospho-GSK3β [113,114]. Curcumin is also involved in the regulation of canonical WNT signaling in ovarian cancer, as combined treatment with 5-aza-2′-deoxycytidine significantly inhibited β-catenin protein expression [119]. Moreover, curcumin treatment suppressed the growth of colon cancer cells in the mouse model and an antitumor effect was mediated by the downregulation of β-catenin and TCF4 via the downregulation of miR-130a [117]. Marjaneh et al. [118], observed that curcumin in combination with 5-flurouracil inhibited cell growth and the invasive behavior of CRC cells through the modulation of the WNT/β-catenin pathway, as GSK-3β phosphorylation decreased significantly after curcumin-5-flurouracil treatment [118]. The safety and efficacy of curcumin in association with 5-flurouracil in colon cancer are evaluated in phase I of the clinical trial (NCT02724202). Curcumin treatment has also been found to be effective in bladder cancer, as it effectively decreased, in a dose-dependent manner, the levels of β-catenin, vimentin and N-cadherin, and significantly increased E-cadherin expression [231]. Anticancer properties of curcumin have also been observed in gastric cancer, where it significantly suppressed the levels of WNT3a, LRP6, β-catenin, C-myc, and survivin, and inhibited the xenograft growth in vivo [116].

To date, clinical trials conducted have demonstrated the safety of curcumin and its derivatives; however, the most commonly reported side effects are mild and predominantly affect the gastrointestinal system [234]. Moreover, various studies have examined the behavior of curcumin in the body, including its distribution, absorption, metabolism and elimination. These studies have found that curcumin is quickly metabolized, poorly absorbed and rapidly eliminated from the body. The lipophilic nature, low stability in aqueous media and low systemic bioavailability of orally administered curcumin are the major drawbacks of its potential use as anticancer drugs [235]. Several delivery systems, such as liposomes, nanoparticles, micelles and phospholipid complexes, have been suggested to enhance the pharmacokinetic properties of curcumin for cancer therapy. For instance, micelles and phospholipid complexes can improve the gastrointestinal absorption of curcumin, resulting in its higher plasma levels [236].

### 4.2. Terpenoids

#### 4.2.1. Lupeol

Lupeol (3-β)-Lup-20(29)-en-3-ol) (LUP) is a pentacyclic triterpenoid found present in fruits (e.g., strawberries, mangos, red grapes and figs), vegetables (e.g., cucumbers, white cabbage, pepper and tomatoes) and medicinal plants (e.g., licorice and *Emblica officinalis*) [237]. Lupeol exhibits several pharmacological activities, including anticancer, antioxidant, anti-inflammatory and antimicrobial properties. Various studies have indicated that lupeol has remarkable potential in preventing and treating different types of cancer, including breast cancer [238], oral cancer [239], lung cancer [240], liver cancer [241], osteosarcoma [242], colorectal cancer [243], bladder cancer [244] and melanoma [157,245]. Some studies suggest that lupeol may be effective as a novel therapeutic option for melanoma patients. Bociort et al. [245] revealed that lupeol exhibited dose-dependent cytotoxic activity, induced apoptosis and inhibited cell migration in A375 and RPMI-7951 malignant melanoma cells [245]. The viability of melanoma cell lines exhibiting high WNT/β-catenin activity (Mel 928 and Mel 1241) was significantly diminished after treatment with lupeol, whereas no impact was observed in cells that lacked constitutively active WNT/β-catenin signaling (Mel 1011) [157]. It has been found that the administration of lupeol has produced a dose-dependent reduction in the levels of β-catenin and WNT target genes, namely coding the region determinant-binding protein (CRD-BP), MITF and CCND1, which was in contradiction to Mel 1011 melanoma cells. Furthermore, the administration of lupeol to a nude mouse model with implanted Mel 928 cells demonstrated a substantial reduction in tumor growth and a decreased expression of c-MYC and CCND1. Additionally, immunohistochemical analysis of the tumors revealed a diminished level of nuclear β-catenin in Mel 928-implanted tumors compared to Mel 1011-implanted tumors, where no alteration of β-catenin localization was observed [157]. These findings suggest that treatment with lupeol may have therapeutic potential in reducing tumor growth and suppressing the expression of oncogenes. Lupeol treatment decreased nuclear β-catenin levels in cells with activated WNT/β-catenin signaling while increasing its cytoplasmic levels. This suggests that lupeol may prevent β-catenin from translocating to the nucleus. Furthermore, the efficiency of lupeol was hindered when WNT signaling was suppressed, and no significant decrease in the number of colonies was observed in the colony formation assay [157]. Saleem et al. [246] also highlighted the potential of lupeol as a therapeutic agent for melanoma treatment, as a significant reduction in the viability of 451Lu and WM35 melanoma cells upon treatment with lupeol was obtained. It has been found that lupeol downregulated BCL2 and upregulated BAX, caused cell cycle arrest in the G_1_-S phase, activated caspase-3 and decreased the expression of cyclin D1, cyclin D2 and CDK2. Moreover, lupeol significantly reduced 451Lu tumor growth in athymic nude mice and modulated the expression of proliferation markers, apoptotic markers and cell cycle regulatory molecules in tumor xenografts [246]. It has also been revealed that lupeol inhibited proliferation and migration, and induced the apoptosis of colorectal cancer cells, which was associated with a decreased expression of β-catenin, TCF4, and β-catenin target genes such as c-Myc and cyclin D1. Moreover, it has been demonstrated that lupeol disturbed stemness in colon cancer cells by regulating Nestin or β-catenin [158]. Similarly, in hepatocellular carcinoma lupeol also induced apoptosis and modulated WNT/β-catenin signaling by the decrease in GSK-3β phosphorylation [159]. It has also been observed that lupeol treatment by targeting β-catenin inhibited the growth of chemoresistant Du145 prostate cancer cells either alone or in combination with Enzalutamide, the second-generation potent androgen receptor antagonist [160].

Despite the therapeutic potential of lupeol, its development as a pharmaceutical drug has been limited by its poor solubility, low bioavailability and inadequate drug delivery [247]. Therefore, numerous approaches have been developed, e.g., lupeol-loaded PEGylated liposomes [248], chitosan-gelatin hydrogel films [249], solid lipid nanoparticles (SLN) [250] and gold nanoparticles [251], to potentiate both the bioavailability and pharmacokinetics of lupeol.

#### 4.2.2. Alantolactone

Alantolactone (ALT) is one of the sesquiterpene lactones (STLs) extracted from *Inula helenium* L. root (elecampane). It is found in East Asia, Europe and North America. ALT can also be derived from *Inula japonica*, *Aucklandia lappa*, *Inula racemosa*, *Inula royleana*, *Rudbeckia subtomentosa* and *Radix inulae* [252]. It has therapeutic potential for treating various diseases such as asthma, bronchitis, tuberculosis and chronic enterogastritis [252,253]. Moreover, in vitro studies have demonstrated that this compound suppresses the growth of numerous cancer types, including colorectal cancer [254], squamous cell lung cancer [255], gastric cancer [256], pancreatic cancer [257], breast cancer [258] and melanoma [161]. ALT has the potential for cancer treatment as it can effectively reduce inflammation and inhibit tumor growth by regulating abnormal signaling pathways in cancer cells [252,253]. ALT inhibited the viability, migration and invasion of A375 and B16 melanoma cells while promoting their apoptosis [161]. It has been suggested that ALT may have therapeutic potential for treating melanoma by suppressing the WNT/β-catenin signaling pathway, as ALT treatment significantly reduced the expression of β-catenin and its downstream effector c-MYC. Moreover, ALT inhibited GSK3β phosphorylation, and GSK3β has been indicated as a key ALT target in melanoma [161]. ALT has effectively modulated WNT/β-catenin signaling in osteosarcoma by the inhibition of GSK3β phosphorylation and the subsequent reduction in β-catenin. ALT treatment resulted in the apoptosis of osteosarcoma cells and restrained the tumor growth and metastasis of osteosarcoma cells in a xenograft model in vivo. Moreover, the combination of ALT and WNT/β-catenin inhibitor (KYA1197K) resulted in a synergistic effect on inhibiting the proliferation, migration and invasion of osteosarcoma cells [162].

However, due to their low water solubility, the absorption and bioavailability of alantolactone is limited [259]. To improve the bioavailability of ALT, nanostructured carriers have been developed, and micellar nanodrugs could prolong the circulation time [260].

### 4.3. Alkaloids

#### Tryptanthrin

Tryptanthrin (indolo [2,1-b] quinazolin-6,12-dione), an indole quinazoline alkaloid, is a naturally occurring chemical compound found in various Chinese medicinal plants, including *Strobilanthes cusia*, *Polygonum tinctorium*, *Isatis tinctoria* [261] and *Wrightia tinctoria* [262]. This compound has gained significant attention as a promising therapeutic agent due to its structural simplicity, ease of synthesis and diverse range of biological properties (antifungal, antiprotozoal, antioxidant, antimicrobial, anti-inflammatory, antiparasitic, antiallergic) [263]. Moreover, the anticancer activity of tryptanthrin has also been reported in leukemia [264,265], breast cancer [266,267], colorectal adenocarcinoma [268], non-melanoma skin cancer [269] and melanoma [163,262]. Tryptanthrin has been suggested as a therapeutic agent for the treatment of melanoma based on the significant cytotoxicity of tryptanthrin towards A375 melanoma cells, with no effects on normal skin fibroblasts or epithelial cells (HEMA-LP) [163,262]. It has been found that tryptanthrin, the constituent of the most active extract of *Wrightia tinctoria* leaves, has efficiently downregulated the oncogenic BRAF in A375 cells and the phosphorylation of ERK1/2, and induced by phorbol-12-myristate-13-acetate (PMA) in a concentration-dependent manner. Additionally, tryptanthrin has downregulated β-catenin, completely abolished MITF-M expression [163,262] and inhibited the expression of AKT and NF-κB [260]. In vivo studies on non-obese diabetic severe combined immunodeficiency mice (NOD-SCID) have shown that tryptanthrin (120 mg/kg) treatment resulted in a remarkable reduction in implanted tumor growth [262] and prevented the metastasis of melanoma cells in distant organs in vivo, and a reduction in the metastatic nodules has been observed in lung tissues of a B16F10 tail vein metastasis model [163]. Moreover, it has been observed that tryptanthrin reduced melanoma metastasis and angiogenesis by a decrease in matrix metalloproteinase 9 (MMP-9) and VEGF, respectively [262]. The wound healing assay demonstrated a dose-dependent inhibition of the melanoma cell migration after tryptanthrin treatment. The evaluation of the expression status of β-catenin in melanoma cell lines revealed the accumulation of cytoplasmic β-catenin levels and unaltered nuclear β-catenin levels, suggesting that tryptanthrin can block the nuclear translocation of β-catenin [163]. The clinical use of tryptanthrin is limited by poor solubility and low bioavailability. It has been found that the solubility of tryptanthrin can be improved by encapsulation in nanoparticles, e.g., nanostructured lipid carriers (NLCs), solid lipid nanoparticles (SLNs), lipid emulsions (LEs) and polycaprolactone-based nanoparticles (NPs) [270,271].

**Figure 2:**
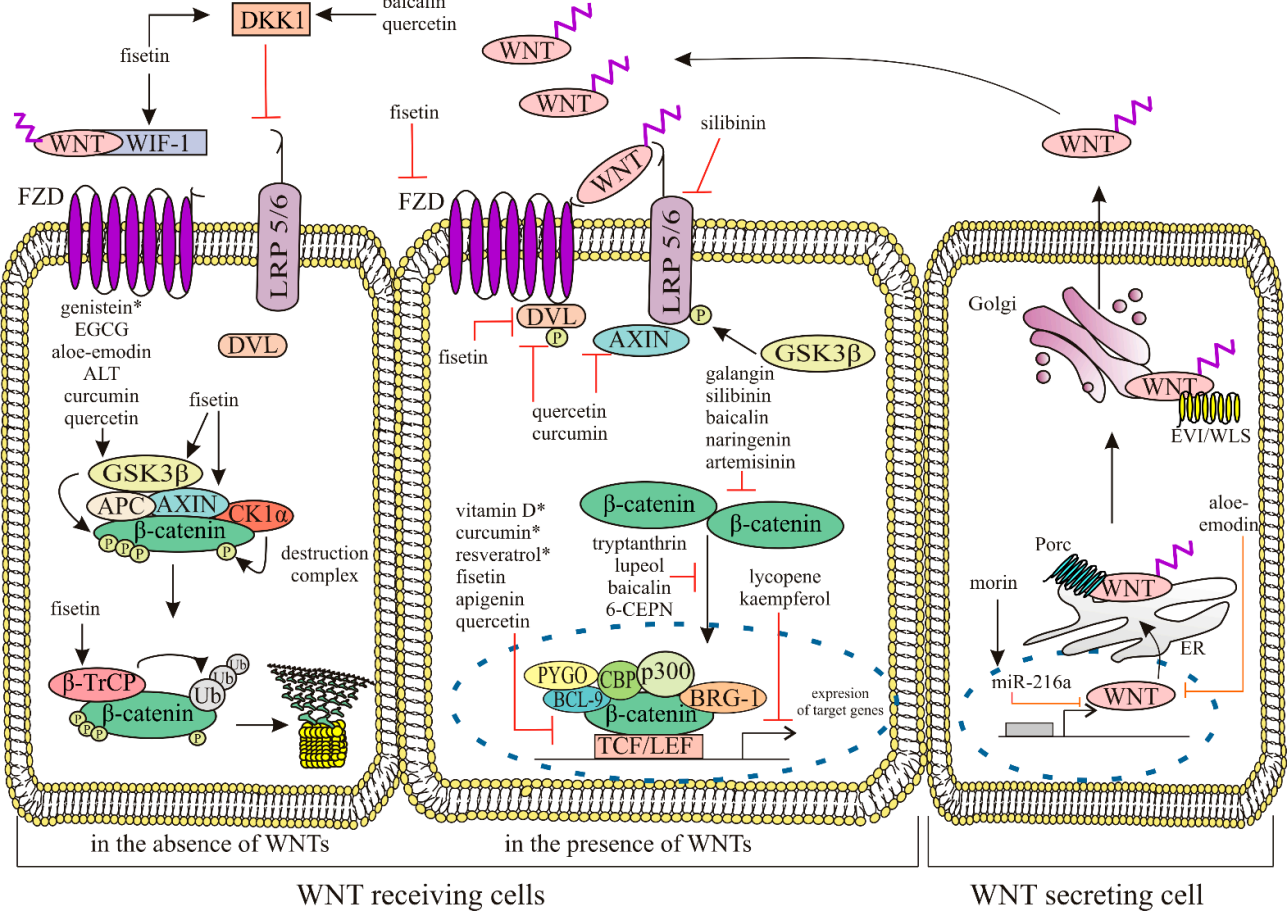
Schematic representation of the modulation of WNT/β-catenin signaling pathway by natural compounds in cancers. The nucleus is indicated by a dashed circle, black arrows mark the stimulatory effect and the inhibitory effect is marked by red bar-headed arrows. (* an agent in the clinical trial). ALT, alantolactone, APC, adenomatous polyposis coli; AXIN, axis inhibition protein; BCL9, B-cell lymphoma 9; BRG1, Brahma-related gene-1; CBP, CREB-binding protein; CK1, casein kinase 1; DKK, Dickkopf; DVL, disheveled protein; EGCG, epigallocatechin-3-gallate; EVI/WLS, Evenness interrupted/Wntless; FZD, frizzled; GSK3β, glycogen synthase kinase 3β; LRP, lipoprotein receptor-related protein; Pygo, Pygopus; TCF/LEF, T-cell factor/Lymphoid enhancer factor; Ub; Ubiquitin; WIF1, WNT inhibitory factor 1; β-TrCP, β-transducin repeat-containing protein.

As demonstrated, natural compounds can affect the canonical WNT signaling pathway in various cancers at various stages, and their mode of action is briefly summarized in Figure 2.

## 5. Conclusions

A WNT/β-catenin signaling pathway is crucial for cancer growth and progression; thus, targeting this signaling cascade might be an effective treatment strategy. However, the canonical WNT signaling is highly complex and context-dependent in cancers. Therefore, the effective modulation of the WNT pathway in cancers represents a significant challenge and remains an area of active research. Synthetic agents targeting WNT/β-catenin signaling frequently exhibit severe side effects. A variety of natural compounds that belong to flavonoids, anthraquinones, terpenoids, alkaloids, and curcuminoids are investigated in preclinical studies and clinical trials for cancer patients. Their usage is attributed to their health benefits, reduced toxicity and side effects in comparison to synthetic agents. More research is needed to better understand the mechanisms of action of natural compounds, the optimal dosing and absorption, and potential side effects before they can be widely used in clinical practice.

## Figures and Tables

**Table 1:** Overview of current and past clinical trials evaluating the activity of synthetic agents targeting WNT pathway in various cancer types (https://clinicaltrials.gov) as of 12 July 2024.

Compound	Mechanism of Action/Target	Trial Identifier (Phase)	Cancer Type	Refs.
LGK974 in monotherapy or with PDR001	porcupine inhibitor	NCT01351103 (phase 1/not recruiting)	melanoma, TNBC pancreatic cancer, BRAF mutant, CRC, cervical SCC, ESCC, lung SCC	[46,47,48]
LGK974 with LGX818 and cetuximab		NCT02278133 (phase 1 and 2/completed)	BRAF-mutant CRC	
ETC-1922159 in monotherapy or with pembrolizumab	porcupine inhibitor	NCT02521844 (phase 1/not recruiting)	CRC	[49]
CGX1321	porcupine inhibitor	NCT03507998 (phase 1)	BDC, CRC, EC, GAC, GIC, HCC, PDAC	[50]
CGX1321 in monotherapy or with pembrolizumab or encorafenib + cetuximab)		NCT02675946 (phase 1)	solid tumors, GISTs	[51]
RXC004 in monotherapy and with pembrolizumab	porcupine inhibitor	NCT04907851 (phase 2/completed)	advanced solid tumors	[52,53]
RXC004 in monotherapy and with nivolumab		NCT03447470 (phase 1/active)	solid tumors	
		NCT04907539 (phase 2/recruiting)	patients with RNF43 or RSPOs aberrated MSS CRC	
XNW7201		NCT03901950 (phase 1/completed)	advanced solid tumors	N/A
OMP-131R10	RSPO inhibitor	NCT02482441 (phase 1/completed)	CRC	[54]
OMP-18R5 (vantictumab)	inhibitors of FZDs	NCT01345201 (phase 1/completed)	solid tumors	[55]
OMP-18R5 with docetaxel		NCT01957007 (phase 1/completed)	solid tumors	
OMP-18R5 with Nab-P and gemcitabine		NCT02005315 (phase 1/completed)	pancreatic cancer	
OMP-18R5 with paclitaxel		NCT01973309 (phase 1/completed)	BC	[56]
OMP-54F28 (ipafricept)	FZD8 decoy receptor for WNT ligands	NCT01608867 (phase 1/completed)	solid tumors	[57,58]
OMP-54F28 with sorafenib		NCT02069145 (phase 1/completed)	HCC	
OMP-54F28 with paclitaxel and carboplatin		NCT02092363 (phase 1/completed)	OC	
OMP-54F28 with Nab-P and gemcitabine		NCT02050178 (phase 1/completed)	pancreatic cancer	
OTSA101	chimeric humanized mAb against FZD10	NCT01469975 (phase 1/terminated)	SS	[59,60]
		NCT04176016 (phase 1/terminated)	relapsed or refractory SS	
PRI-724	β-catenin/ CBP inhibitor	NCT01302405 (phase 1/completed)	CRC	[61]
PRI-724 with gemcitabine		NCT01764477 (phase 1/completed)	advanced or metastatic PDAC	[62,63]
PRI-724		NCT01606579 (phase 1 and 2/completed)	AML	[64]
CWP232291	β-catenin	NCT03055286 (phase 1& phase 2)	AML	[65]
		NCT01398462 (phase 1/completed)	AML, CMML MDS	
CWP232291 in monotherapy or with lenalidomide and dexamethasone		NCT02426723 (phase 1/completed)	MM	[66]
DKN-01 in monotherapy or with sorafenib	DKK1 inhibitor	NCT03645980 (phase 1 and 2)	HCC	N/A
DKN-01 in monotherapy or with atezolizumab		NCT04166721 (phase 1 and 2/recruiting)	metastatic EC metastatic GC	N/A
DKN-01 in monotherapy or with docetaxel		NCT03837353 (phase 1 and 2/terminated)	PC	[67]
DKN-01		NCT01457417 (phase 1/completed)	MM, solid tumors, NSCLC	[68]
DKN-01 in monotherapy or with paclitaxel		NCT03395080 (phase 2/completed)	endometrial cancer, OC, uterine cancer, carcinosarcoma	[69]
DKN-01 in monotherapy or with paclitaxel or pembrolizumab		NCT02013154 (phase 1/completed)	EC, GEJA, GOJ, SCC, GAC	[70]
DKN-01with lenalidomide/ dexamethasone		NCT01711671 (phase 1/completed)	MM	N/A
DKN-01 with gemcitabine and cisplatin		NCT02375880 (phase 1/completed)	ICCs, ECCs, CCA	[71,72]
DKN-01 with FOLFIRI/FOLFOX and bevacizumab		NCT05480306 (phase 2/completed)	CRC	[73]
DKN-01 with tislelizumab ± CAPOX		NCT04363801 (phase 2/active)	GC, GAC, GOJ	[74]
DKN-01		NCT04681248 (available)	EC, GEJA, GOJ, SCC, GAC, UC, OC, CS, GC	N/A
DKN-01 in monotherapy or with paclitaxel		NCT03818997 (phase 2/withdrawn)	EC, BTC, GOJ, HPB cancer	N/A
DKN-01 with pembrolizumab		NCT05761951 (phase 2/recruiting)	endometrial cancer	[75]
DKN-01 with nivolumab		NCT04057365 (phase 2/terminated)	BTC	N/A
Foxy-5	WNT5A mimicking peptide	NCT02020291 (phase 1/completed)	MBC, mCRC, mPC	[76]
		NCT02655952 (phase 1/completed)		
		NCT03883802 (phase 2/not recruiting)	CRC	
UC-961 (cirmtuzumab)	Anti-ROR1 Monoclonal Antibody	NCT02860676 (phase 1/completed)	CLL	[77]
		NCT02222688 (phase 1/completed)		
		NCT02776917 (phase 1/completed)	BC	[78]
UC-961 with ibrutinib		NCT03088878 (phase 1 and phase 2/ not recruiting,)	B-CLL, SLL, MCL, MZL	[79]
		NCT05431179 (phase 3/withdrawn)	LPDs, LD, NHL	
sulindac	DVL	NCT00245024 (phase 1/completed)	BC	[80]
		NCT04542135 (phase 2/recruiting)		N/A
		NCT01856322 (phase 2/terminated)	CRC	N/A
		NCT00368927 (phase 2/completed)	NSCLC	[81]
		NCT00062023 (phase 2/terminated)	CRC	N/A
BNC101	LGR5	NCT02726334 (phase 1/terminated)	CRC	[82]
pyrvinium pamoate	CK1 activation	NCT05055323 (phase 1/recruiting)	PDAC	N/A
tegavivint	β-catenin	NCT03459469 (phase 1/completed)	DTs	[83]
		NCT04874480 (phase 1/not recruiting)	recurrent leukemia	N/A
		NCT04851119 (phase 1 and 2/recruiting)	solid tumors	N/A
tegavivint with osimertinib		NCT04780568 (phase 1/recruiting)	NSCLC	N/A
E7449 (2X-121)	TNK1/2	NCT03878849 (phase 2/recruiting)	OC	[84]
E7449 in monotherapy or with temozolomide or carboplatin and paclitaxel		NCT01618136 (phase 1 and 2/completed)	solid tumors	
ST316	inhibition of BCL9 and β-catenin interaction	NCT05848739 (phase 1/recruiting)	BC, pancreatic cancer, NSCLC, SS, CRC, HCC, TNBC, OC skin cancer, melanoma, CCA	[85,86]
niclosamide	FZD1 and BCL-9 inhibition, c-JUN upregulation	NCT02687009 (phase 1/terminated)	CRC	[87]
	FZD1 inhibition	NCT02519582 (phase 1/unknown status)	CRC	[88]
		NCT05188170 (phase 2/recruiting)	AML	N/A
niclosamide with enzalutamide		NCT03123978 (phase 1/completed)	mPC, recurrent PC	N/A
		NCT02532114 (phase 1/completed)	mPC	[89]
niclosamide with abiraterone acetate and prednisone		NCT02807805 (phase 2/active)	mPC	N/A
riluzole	GRM1 inhibition	NCT00866840 (phase 2/completed)	melanoma	[90]
		NCT01303341 (phase 1/not recruiting)	malignant solid neoplasms, recurrent melanoma cutaneous melanoma	

AML, acute myeloid leukemia; B-CLL, B-cell chronic lymphocytic leukemia; BC, breast cancer; BDC, bile duct cancer; BTC, biliary tract cancer; CC, cervical cancer; CCA, cholangiocarcinoma; CLL, chronic lymphocytic leukemia; CMML, chronic myelomonocytic leukemia; CRC, colorectal cancer; CS, carcinosarcoma; DTs, desmoid tumors; EC, esophageal cancer; ECCs, extrahepatic cholangiocarcinomas; ESCC, esophageal squamous-cell carcinomas; GAC, gastric adenocarcinoma; GC, gastric cancer; GEJA, gastroesophageal junction adenocarcinoma; GIC, gastrointestinal cancer; GISTs, gastrointestinal stromal tumors; GOJ, gastrooesophageal junction; GRM1, glutamate receptor 1; HBP1, HMG-box transcription factor 1; HCC, hepatocellular carcinoma; HPB, hepatobiliary; ICCs, intrahepatic cholangiocarcinomas; JMJD2, Jumonji C domain-containing 2; LD, lymphatic disease; LGX818, encorafenib; LPDs, lymphoproliferative disorders; mAb, monoclonal antibody; MBC, metastatic breast cancer; MCL, mantle cell lymphoma; mCRC, Metastatic colorectal cancer; MDS, myelodysplastic syndrome; MM, multiple myeloma; mPC, metastatic prostate cancer; MSS, microsatellite stable; MZL, marginal zone lymphoma; N/A, not available; Nab-P, nab-paclitaxel; NHL, non-Hodgkin’s lymphoma; NSCLC, non-small-cell lung cancer; OC, ovarian cancer; PC, prostate cancer; PDAC, pancreatic adenocarcinoma; PDR001, spartalizumab; RNF43, ring finger protein 43; RSPOs, R-spondins; SCC, squamous cell carcinoma; SLL, small lymphocytic lymphoma; SS, synovial sarcoma; TC, thyroid carcinoma; TNBC, triple-negative breast cancer; UC, uterine cancer.

**Table 2:** Overview of clinical trials and preclinical studies evaluating the activity of natural compounds targeting WNT pathway in various cancer types (https://clinicaltrials.gov), as of 12 July 2024.

Compound	Natural Sources	Mechanism of Action/Target	Trial Identifier (Phase)	Cancer Type	Refs.
curcumin with 5-fluorouracil	turmeric	β-catenin inhibition	NCT02724202 (early phase 1)	CRC	N/A
curcumin with irinotecan			NCT01859858 (phase 1/completed)		[107]
curcumin with celecoxib			NCT00295035 (phase 3)		[108]
curcumin with capecitabine and radiation			NCT00745134 (phase 2/terminated)	CRC	[109]
curcumin		destabilization of the WNT-FZDs-LRP6 complex	preclinical	melanoma	[110]
		inhibition of β-catenin/DVL		BC	[111]
		GSK-3β activation			[112]
				NSCLC, MB	[113,114]
		suppression of β-catenin, vimentin and N-cadherin, stimulation of E-cadherin		BC	[115]
		suppression of WNT3a, LRP6, β-catenin, C-myc, and survivin		GC	[116]
		downregulation of β-catenin and TCF4		CRC	[117]
curcumin with 5-fluoracil		GSK-3β activation		CRC	[118]
curcumin with 5-aza-2′ deoxycytidine		β-catenin inhibition		OC	[119]
genistein with FOLFOX or FOLFOX-Avastin	beans, soy, lentils, peas	GSK-3β activation	NCT01985763 (phase 1 and 2/completed)	CRC	[120,121]
resveratrol	grapes, wine, peanuts, soy	inhibition of β-catenin/TCF	NCT00256334 (phase 1/completed)	CRC	[122]
SRT501 (micronized resveratrol)			NCT00920803 (phase 1/completed)		[123]
dietary grape-derived low-dose resveratrol			NCT00578396 (phase 1/withdrawn)	CRC	N/A
vitamin D	mushrooms, fish liver oils, cheese, beef liver, eggs, dark chocolate, milk, yogurt, fat spreads, orange juice, breakfast grains, plant-based beverages	inhibition of β-catenin/TCF induction of E-cadherin expression induction of DKK1 expression	NCT00399607 (adjunct study completed)	colorectal adenomatous polyps	N/A
			NCT02603757 (completed)	CRC	N/A
EGCG	cocoa products, tea, pome fruits	GSK-3β activation	preclinical	LC, GC, CRC, skin cancer	[124,125,126,127]
		upregulation of HBP1		BC	[128]
apigenin	celery seeds, spinach, parsley, marjoram, Italian oregano, sage, chamomile, pistachio	inhibition of β-catenin/TCF/LEF	preclinical	CRC	[129,130]
		lncRNA H19		HCC	[131]
baicalin	Baikal skullcap, Indian trumpet tree	induction of DKK1 expression	preclinical	colon cancer	[132]
		inhibition of β-catenin and AXIN2 and stimulation of GSK-3β expression		osteosarcoma	[133]
		suppression of β-catenin nuclear translocation		CC	[134]
		β-catenin inhibition		T-ALL	[135]
				BC	[136]
galangin	honey, lesser galangal	β-catenin inhibition	preclinical	CRC, liver cancer	[137]
galangin with berberine		downregulation of WNT3a and β-catenin		EC	[138]
silibinin	seeds of thistle and artichoke	suppression of LRP6	preclinical	PC, BC	[139]
		β-catenin inhibition		CRC	[140]
kaempferol	spinach, kale, dill, chives, tarragon	suppression of β-catenin/TCF transcriptional activity	preclinical	CRC	[141]
		reduction in JMJD2C/β-catenin signaling			[142]
		β-catenin inhibition		Rb	[143]
lycopene	tomatoes, apricots, melons, papayas, grapes, peaches, watermelons, cranberries	activation of GSK-3β; increase APC and β-TrCP	preclinical	GC	[144]
lycopene with quinacrine		reduction in TCF/LEF reporter activity		BC	[145]
naringenin	grapes, oranges, bergamots, lemons	β-catenin inhibition	preclinical	OC	[146]
6-CEPN		β-catenin degradation, inhibition of nuclear β-catenin translocation	preclinical	HCC	[147]
artemisinin dihydroartemisinin, artesunate	wheat wormwood	β-catenin inhibition	preclinical	NSCLC	[148]
quercetin	onion, asparagus, berries	activation of GSK-3β; inhibition of β-catenin/TCF and WNT-FZDs-LRP6; increase of E-cadherin and DKK-1/2/3	preclinical	CRC, TC, melanoma, TNBC	[110,149,150,151]
fisetin	strawberry, apple, persimmon, grape, onion, cucumber	activation of DKK1; WIF; GSK-3β; inhibition of β-catenin/TCF	preclinical	melanoma, colon cancer	[152,153]
morin	figs, chestnut, jack fruit, red wine, seaweed, tea, coffee, cereal grains	inhibition of WNT3A	preclinical	melanoma	[154]
aloe emodin	coffee senna, Chinese rhubarb, aloe, tuber fleeceflower	inhibition of WNT3a and p-GSK3-β	preclinical	melanoma	[155]
		reduction in WNT2 and β-catenin mRNA		PC	[156]
lupeol	cabbage, pepper, cucumber, tomato, olive, fig, mango, strawberry, red grapes, American ginseng, shea butter	blocking nuclear β-catenin translocation	preclinical	melanoma	[157]
		inhibition of β-catenin/TCF4		colon cancer	[158]
		activation of GSK-3β		HCC	[159]
		inhibition of β-catenin		PC	[160]
alantolactone	elecampane	activation of GSK-3β	preclinical	melanoma	[161]
				osteosarcoma	[162]
tryptanthrin	assam indigo, Chinese indigo, woad	inhibition of nuclear β-catenin translocation	preclinical	melanoma	[163]

BC, breast cancer; CC, cervical cancer; CRC, colorectal cancer; EC, esophageal cancer; EGCG, epigallocatechin-3-gallate; GC, gastric cancer; HBP1, HMG-box transcription factor 1; HCC, hepatocellular carcinoma; JMJD2, Jumonji C domain-containing 2; LC, lung cancer; MB, medulloblastoma; N/A, not available; NSCLC, non-small-cell lung cancer; OC, ovarian cancer; PC, prostate cancer; T-ALL, T-cell acute lymphoblastic leukemia; TC, thyroid carcinoma; TNBC, triple-negative breast cancer; 6-CEPN, 6-C-(E-phenylethenyl)naringenin.

## Data Availability

This is a review and no data were used for the research described in this article.

## Abbreviations

AKT, protein kinase B signaling; ALT, alantolactone; AML, acute myeloid leukemia; APC, adenomatous polyposis coli; AXIN, axis inhibition protein; BCL9, B-cell lymphoma 9; BRG1, Brahma-related gene-1; CBP, CREB-binding protein; CCND1, cyclin D1; 6-CEPN, 6-C-(E-phenylethenyl)naringenin; CER, Cerberus protein; CK1, casein kinase 1; COX2, cyclooxygenase; CRD-BP, coding region determinant binding protein; CRD, cysteine-rich domain; DKK, Dickkopf; DTIC, dacarbazine; DVL, disheveled protein; EGCG, epigallocatechin-3-gallate; EVI/WLS, Evenness interrupted/Wntless; FGF, fibroblast growth factor; FRA1, FOS-related antigen 1; FZDs, frizzled receptors; GPCRs, G-protein coupled receptors; GRM1, metabotropic glutamate receptor 1; GSK3β, glycogen synthase kinase 3β; HBP1, HMG-box transcription factor 1; HCC, hepatocellular carcinoma, ICIs, immune checkpoint inhibitors; IFN, interferon; IL, interleukin; JMJD2, Jumonji C domain-containing 2; KRM, Kremen; LEs, lipid emulsions; LGR, leucine-rich repeat-containing G-protein-coupled receptor; LRP, lipoprotein receptor-related protein; LUP, lupeol; MITF, microphthalmia-associated transcription factor; MMP-9, matrix metalloproteinase 9; ncRNA, non-coding RNAs; NLCs, nanostructured lipid carriers; NLS, nuclear localization sequence; NPC, nuclear pore complex; NPs, nanoparticles; NSCLC, non-small-cell lung cancer; PI3K, phosphoinositol-3-kinase; PMA, phorbol-12-myristate-13-acetate; PYGO, Pygopus; RNF43, ring finger protein 43; RSPOs, R-spondins; sFRPs, secreted frizzled-related proteins; SLNs, solid lipid nanoparticles; SP1, specificity protein 1; TCF/LEF, T-cell factor/Lymphoid enhancer factor; TNBC, triple-negative breast cancer; TNKS, tankyrase; VEGF, vascular endothelial growth factor; WIF1, WNT inhibitory factor 1; WISE, WNT modulator in surface ectoderm; WISP1, WNT1-inducible-signaling pathway protein 1; ZNF3, zinc and ring finger 3; β-TrCP, β-transducin repeat-containing protein.

## References

[1] Nusse R., Clevers H. (2017). Wnt/β-Catenin signaling, disease, and emerging therapeutic modalities. Cell.

[2] Ring A., Kim Y.M., Kahn M. (2014). Wnt/β-catenin signaling in adult stem cell physiology and disease. Stem Cell Rev. Rep..

[3] Galluzzi L., Spranger S., Fuchs E., López-Soto A. (2019). WNT signaling in cancer immunosurveillance. Trends Cell. Biol..

[4] Liu J., Xiao Q., Xiao J., Niu C., Li Y., Zhang X., Zhou Z., Shu G., Yin G. (2022). Wnt/β-catenin signalling: Function, biological mechanisms, and therapeutic opportunities. Sig. Transduct. Target. Ther..

[5] Liu D., Chen L., Zhao H., Vaziri N.D., Ma S.C., Zhao Y.Y. (2019). Small molecules from natural products targeting the Wnt/β-catenin pathway as a therapeutic strategy. Biomed. Pharmacother..

[6] Yang P., Zhu Y., Zheng Q., Meng S., Wu Y., Shuai W., Sun Q., Wang G. (2022). Recent advances of β-catenin small molecule inhibitors for cancer therapy: Current development and future perspectives. Eur. J. Med. Chem..

[7] Werner J., Boonekamp K.E., Zhan T., Boutros M. (2023). The Roles of Secreted Wnt Ligands in Cancer. Int. J. Mol. Sci..

[8] Raslan A.A., Yoon J.K. (2019). R-spondins: Multi-mode WNT signaling regulators in adult stem cells. Int. J. Biochem. Cell Biol..

[9] Martin-Orozco E., Sanchez-Fernandez A., Ortiz-Parra I., Ayala-San Nicolas M. (2019). WNT signaling in tumors: The way to evade drugs and immunity. Front. Immunol..

[10] Hao H.X., Xie Y., Zhang Y., Zhang O., Oster E., Avello M., Lei H., Mickanin C., Liu D., Ruffner H. (2012). ZNRF3 promotes Wnt receptor turnover in an R-spondin-sensitive manner. Nature.

[11] Koo B.K., Spit M., Jordens I., Low T.Y., Stange D.E., Van De Wetering M., van Es J.H., Mohammed S., Heck A.J., Maurice M.M. (2012). Tumour suppressor RNF43 is a stem-cell E3 ligase that induces endocytosis of Wnt receptors. Nature.

[12] Komiya Y., Habas R. (2008). Wnt signal transduction pathways. Organogenesis.

[13] O’Connell M.P., Weeraratna A.T. (2009). Hear the Wnt Ror: How melanoma cells adjust to changes in Wnt. Pigment Cell Melanoma Res..

[14] Larue L., Delmas V. (2006). The WNT/Beta-catenin pathway in melanoma. Front. Biosci..

[15] Buechling T., Boutros M. (2011). Wnt signaling signaling at and above the receptor level. Curr. Top. Dev. Biol..

[16] Ackers I., Malgor R. (2018). Interrelationship of canonical and non-canonical Wnt signalling pathways in chronic metabolic diseases. Diab. Vasc. Dis. Res..

[17] Qin K., Yu M., Fan J., Wang H., Zhao P., Zhao G., Zeng W., Chen C., Wang Y., Wang A. (2023). Canonical and noncanonical Wnt signaling: Multilayered mediators, signaling mechanisms and major signaling crosstalk. Genes Dis..

[18] Pascual-Vargas P., Salinas P.C. (2021). A role for frizzled and their post-translational modifications in the mammalian central nervous system. Front. Cell. Dev. Biol..

[19] Gajos-Michniewicz A., Czyz M. (2020). WNT signaling in melanoma. Int. J. Mol. Sci..

[20] Anthony C.C., Robbins D.J., Ahmed Y., Lee E. (2020). Nuclear Regulation of Wnt/β-catenin signaling: It’s a complex situation. Genes.

[21] Lu J., Wu T., Zhang B., Liu S., Song W., Qiao J., Ruan H. (2021). Types of nuclear localization signals and mechanisms of protein import into the nucleus. Cell Commun. Signal..

[22] Widlund H.R., Horstmann M.A., Price E.R., Cui J., Lessnick S.L., Wu M., He X., Fisher D.E. (2002). Beta-catenin-induced melanoma growth requires the downstream target Microphthalmia-associated transcription factor. J. Cell Biol..

[23] Yuzhalin A.E. (2024). Redefining cancer research for therapeutic breakthroughs. Br. J. Cancer.

[24] Khan S.U., Fatima K., Aisha S., Malik F. (2024). Unveiling the mechanisms and challenges of cancer drug resistance. Cell Commun. Signal..

[25] Park W.J., Kim M.J. (2023). A new wave of targeting ‘undruggable’ Wnt signaling for cancer therapy: Challenges and opportunities. Cells.

[26] Zhong Z., Virshup D.M. (2020). Wnt signaling and drug resistance in cancer. Mol. Pharmacol..

[27] Zhan T., Rindtorff N., Boutros M. (2017). Wnt signaling in cancer. Oncogene.

[28] Bugter J.M., Fenderico N., Maurice M.M. (2021). Mutations and mechanisms of WNT pathway tumour suppressors in cancer. Nat. Rev. Cancer.

[29] Groenewald W., Lund A.H., Gay D.M. (2023). The role of Wnt pathway mutations in cancer development and an overview of therapeutic options. Cells.

[30] Jung Y.S., Park J.I. (2020). Wnt signaling in cancer: Therapeutic targeting of Wnt signaling beyond β-catenin and the destruction complex. Exp. Mol. Med..

[31] Arozarena I., Bischof H., Gilby D., Belloni B., Dummer R., Wellbrock C. (2011). In melanoma, beta-catenin is a suppressor of invasion. Oncogene.

[32] Kageshita T., Hamby C.V., Ishihara T., Matsumoto K., Saida T., Ono T. (2001). Loss of beta-catenin expression associated with disease progression in malignant melanoma. Br. J. Dermatol..

[33] Chien A.J., Moore E.C., Lonsdorf A.S., Kulikauskas R.M., Rothberg B.G., Berger A.J., Major M.B., Hwang S.T., Rimm D.L., Moon R.T. (2009). Activated Wnt/beta-catenin signaling in melanoma is associated with decreased proliferation in patient tumors and a murine melanoma model. Proc. Natl. Acad. Sci. USA.

[34] Bian J., Dannappel M., Wan C., Firestein R. (2020). Transcriptional regulation of Wnt/β-catenin pathway in colorectal cancer. Cells.

[35] Gajos-Michniewicz A., Czyz M. (2023). WNT/β-catenin signaling in hepatocellular carcinoma: The aberrant activation, pathogenic roles, and therapeutic opportunities. Genes Dis..

[36] Wang S., Zheng Y., Yang F., Zhu L., Zhu X.Q., Wang Z.F., Wu X.L., Zhou C.H., Yan J.Y., Hu B.Y. (2021). The molecular biology of pancreatic adenocarcinoma: Translational challenges and clinical perspectives. Signal. Transduct. Target. Ther..

[37] Rapp J., Jaromi L., Kvell K., Miskei G., Pongracz J.E. (2017). WNT signaling—Lung cancer is no exception. Respir. Res..

[38] Nguyen V.H.L., Hough R., Bernaudo S., Peng C. (2019). Wnt/β-catenin signalling in ovarian cancer: Insights into its hyperactivation and function in tumorigenesis. J. Ovarian Res..

[39] Kovacs D., Migliano E., Muscardin L., Silipo V., Catricalà C., Picardo M., Bellei B. (2016). The role of Wnt/β-catenin signaling pathway in melanoma epithelial-to-mesenchymal-like switching evidences from patients-derived cell lines. Oncotarget.

[40] Delmas V., Beermann F., Martinozzi S., Carreira S., Ackermann J., Kumasaka M., Denat L., Goodall J., Luciani F., Viros A. (2007). Beta-catenin induces immortalization of melanocytes by suppressing p16INK4a expression and cooperates with N-Ras in melanoma development. Genes Dev..

[41] Goodall J., Martinozzi S., Dexter T.J., Champeval D., Carreira S., Larue L., Goding C.R. (2004). Brn-2 expression controls melanoma proliferation and is directly regulated by beta-catenin. Mol. Cell. Biol..

[42] Wang B., Tian T., Kalland K.H., Ke X., Qu Y. (2018). Targeting Wnt/β-catenin signaling for cancer immunotherapy. Trends Pharmacol. Sci..

[43] Sferrazza G., Corti M., Brusotti G., Pierimarchi P., Temporini C., Serafino A., Calleri E. (2020). Nature-derived compounds modulating Wnt/β-catenin pathway: A preventive and therapeutic opportunity in neoplastic diseases. Acta Pharm. Sin. B.

[44] Han J., Lin K., Zhang X., Yan L., Chen Y., Chen H., Liu J., Liu J., Wu Y. (2021). PTEN-mediated AKT/β-catenin signaling enhances the proliferation and expansion of Lgr5+ hepatocytes. Int. J. Biol. Sci..

[45] He J., Han J., Lin K., Wang J., Li G., Li X., Gao Y. (2023). PTEN/AKT and Wnt/β-catenin signaling pathways regulate the proliferation of Lgr5+ cells in liver cancer. Biochem. Biophys. Res. Commun..

[46] Rodon J., Argilés G., Connolly R.M., Vaishampayan U., de Jonge M., Garralda E., Giannakis M., Smith D.C., Dobson J.R., McLaughlin M.E. (2021). Phase 1 study of single-agent WNT974, a first-in-class Porcupine inhibitor, in patients with advanced solid tumors. Br. J. Cancer.

[47] Bagheri M., Tabatabae Far M.A., Mirzaei H., Ghasemi F. (2020). Evaluation of antitumor effects of aspirin and LGK974 drugs on cellular signaling pathways, cell cycle and apoptosis in colorectal cancer cell lines compared to oxaliplatin drug. Fundam. Clin. Pharmacol..

[48] Liu J., Pan S., Hsieh M.H., Ng N., Sun F., Wang T., Kasibhatla S., Schuller A.G., Li A.G., Cheng D. (2013). Targeting Wnt-driven cancer through the inhibition of Porcupine by LGK974. Proc. Natl. Acad. Sci. USA.

[49] Madan B., Ke Z., Harmston N., Ho S.Y., Frois A.O., Alam J., Jeyaraj D.A., Pendharkar V., Ghosh K., Virshup I.H. (2016). Wnt addiction of genetically defined cancers reversed by PORCN inhibition. Oncogene.

[50] Li C., Cao J., Zhang N., Tu M., Xu F., Wei S., Chen X., Xu Y. (2018). Identification of RSPO2 fusion mutations and target therapy using a porcupine inhibitor. Sci. Rep..

[51] Giannakis M., Le D.T., Pishvaian M.J., Weinberg B.A., Papadopoulos K.P., Shen L., Gong J., Li J., Strickler J.H., Zhou A. (2023). Phase 1 Study of WNT Pathway Porcupine Inhibitor CGX1321 and Phase 1b Study of CGX1321 + Pembrolizumab (Pembro) in Patients (Pts) with Advanced Gastrointestinal (GI) Tumors. J. Clin. Oncol..

[52] Phillips C., Bhamra I., Eagle C., Flanagan E., Armer R., Jones C.D., Bingham M., Calcraft P., Edmenson Cook A., Thompson B. (2022). The Wnt Pathway Inhibitor RXC004 Blocks Tumor Growth and Reverses Immune Evasion in Wnt Ligand-dependent Cancer Models. Cancer Res. Commun..

[53] Cook N., Blagden S., Lopez J., Sarker D., Greystoke A., Harris N., Kazmi F., Naderi A., Nintos G., Franco A.O. (2021). 517MO Phase I Study of the Porcupine (PORCN) Inhibitor RXC004 in Patients with Advanced Solid Tumors. Ann. Oncol..

[54] Bendell J., Eckhardt G.S., Hochster H.S., Morris V.K., Strickler J., Kapoun A.M., Wang M., Xu L., McGuire K., Dupont J. (2016). Initial results from a phase 1a/b study of OMP-131R10, a first-in-class anti-RSPO3 antibody, in advanced solid tumors and previously treated metastatic colorectal cancer (CRC). Eur. J. Cancer.

[55] Gurney A., Axelrod F., Bond C.J., Cain J., Chartier C., Donigan L., Fischer M., Chaudhari A., Ji M., Kapoun A.M. (2012). Wnt pathway inhibition via the targeting of Frizzled receptors results in decreased growth and tumorigenicity of human tumors. Proc. Natl. Acad. Sci. USA.

[56] Diamond J.R., Becerra C., Richards D., Mita A., Osborne C., O’Shaughnessy J., Zhang C., Henner R., Kapoun A.M., Xu L. (2020). Phase Ib clinical trial of the anti-frizzled antibody vantictumab (OMP-18R5) plus paclitaxel in patients with locally advanced or metastatic HER2-negative breast cancer. Breast Cancer Res. Treat..

[57] Le P.N., McDermott J.D., Jimeno A. (2015). Targeting the Wnt pathway in human cancers: Therapeutic targeting with a focus on OMP-54F28. Pharmacol. Ther..

[58] Jimeno A., Gordon M., Chugh R., Messersmith W., Mendelson D., Dupont J., Stagg R., Kapoun A.M., Xu L., Uttamsingh S. (2017). A first-in-human phase i study of the anticancer stem cell agent ipafricept (OMP-54F28), a decoy receptor for wnt ligands, in patients with advanced solid tumors. Clin. Cancer Res..

[59] Giraudet A.L., Cassier P.A., Iwao-Fukukawa C., Garin G., Badel J.N., Kryza D., Chabaud S., Gilles-Afchain L., Clapisson G., Desuzinges C. (2018). A first-in-human study investigating biodistribution, safety and recommended dose of a new radiolabeled MAb targeting FZD10 in metastatic synovial sarcoma patients. BMC Cancer.

[60] Sudo H., Tsuji A.B., Sugyo A., Harada Y., Nagayama S., Katagiri T., Nakamura Y., Higashi T. (2022). FZD10-targeted α-radioimmunotherapy with ^225^ Ac-labeled OTSA101 achieves complete remission in a synovial sarcoma model. Cancer Sci..

[61] El-Khoueiry A.B., Ning Y., Yang D., Cole S., Kahn M., Zoghbi M., Berg J., Fujimori M., Inada T., Kouji H. (2013). A phase I first-in-human study of PRI-724 in patients (pts) with advanced solid tumors. J. Clin. Oncol..

[62] McWilliams R.R., Ko A.H., Chiorean E.G., Kwak E.L., Lenz H.-J., Nadler P.I., Wood D.L., Fujimori M., Morita K., Inada T. (2015). A phase Ib dose-escalation study of PRI-724, a CBP/beta-catenin modulator, plus gemcitabine (GEM) in patients with advanced pancreatic adenocarcinoma (APC) as second-line therapy after FOLFIRINOX or FOLFOX. J. Clin. Oncol..

[63] Ko A.H., Chiorean E.G., Kwak E.L., Lenz H.-J., Nadler P.I., Wood D.L., Fujimori M., Inada T., Kouji H., McWilliams R.R. (2016). Final results of a phase Ib dose-escalation study of PRI-724, a CBP/beta-catenin modulator, plus gemcitabine (GEM) in patients with advanced pancreatic adenocarcinoma (APC) as second-line therapy after FOLFIRINOX or FOLFOX. J. Clin. Oncol..

[64] Láinez-González D., Alonso-Aguado A.B., Alonso-Dominguez J.M. (2023). Understanding the Wnt signaling pathway in acute myeloid leukemia stem cells: A feasible key against relapses. Biology.

[65] Lee J.H., Faderl S., Pagel J.M., Jung C.W., Yoon S.S., Pardanani A.D., Becker P.S., Lee H., Choi J., Lee K. (2020). Phase 1 study of CWP232291 in patients with relapsed or refractory acute myeloid leukemia and myelodysplastic syndrome. Blood Adv..

[66] Manasanch E.E., Yoon S.-S., Min C.-K., Kim J.S., Shain K.H., Hauptschein R., Choi J.E., Lee K.-J. (2017). Interim results from the phase 1a/1b dose-finding study of CWP232291 (CWP291) in relapsed or refractory myeloma (RRMM) alone or in combination with lenalidomide and dexamethasone. Blood.

[67] Wise D.R., Schneider J.A., Armenia J., Febles V.A., McLaughlin B., Brennan R., Thoren K.L., Abida W., Sfanos K.S., De Marzo A.M. (2020). International SU2C/PCF Prostate Cancer Dream Team (2020). Dickkopf-1 can lead to immune evasion in metastatic castration-resistant prostate cancer. JCO Precis Oncol..

[68] Edenfield W.J., Richards D.A., Vukelja S.J., Weiss G.J., Sirard C.A., Landau S.B., Ramanathan R.K. (2014). A phase 1 study evaluating the safety and efficacy of DKN-01, an investigational monoclonal antibody (Mab) in patients (pts) with advanced non-small cell lung cancer. J. Clin. Oncol..

[69] Arend R.C., Castro C.M., Matulonis U.A., Hamilton E., Gunderson C.C., Lybarger K.S.S., Goodman H.M., Duska L.R., Mahdi H., ElNaggar A.C. (2020). Dkn-01 treated patients with recurrent epithelial endometrial (EEC) or ovarian (EOC) cancers which harbor Wnt activating mutations have longer progression-free survival and improved clinical benefit. Gynecol. Oncol..

[70] Klempner S.J., Bendell J.C., Villaflor V.M., Tenner L.L., Stein S., Naik G.S., Sirard C.A., Kagey M., Chaney M.F., Strickler J.H. (2020). DKN-01 in combination with pembrolizumab in patients with advanced gastroesophageal adenocarcinoma (GEA): Tumoral DKK1 expression as a predictor of response and survival. J. Clin. Oncol..

[71] Eads J.R., Goyal L., Stein S., El-Khoueiry A.B., Manji G.A., Abrams T.A., Landau S.B., Sirard C.A. (2016). Phase I study of DKN-01, an anti-DKK1 antibody, in combination with gemcitabine (G) and cisplatin (C) in patients (pts) with advanced biliary cancer. J. Clin. Oncol..

[72] Goyal L., Sirard C., Schrag M., Kagey M.H., Eads J.R., Stein S., El-Khoueiry A.B., Manji G.A., Abrams T.A., Khorana A.A. (2020). Phase I and Biomarker Study of the Wnt Pathway Modulator DKN-01 in Combination with Gemcitabine/Cisplatin in Advanced Biliary Tract Cancer. Clin. Cancer Res..

[73] Wainberg Z.A., Boccia R.V., Strickler J.H., Moehler M.H., Sirard C.A., Walsh E.K., Parker E.C., Lee K.-W. (2023). Randomized phase 2 study of DKN-01 plus FOLFIRI/FOLFOX and bevacizumab versus FOLFIRI/FOLFOX and bevacizumab as second-line treatment of advanced colorectal cancer (DeFianCe). J. Clin. Oncol..

[74] Klempner S.J., Sonbol B.B., Wainberg Z.A., Uronis H.E., Chiu V.K., Scott A.J., Iqbal S., Tejani M.A., Stilian M.C., Thoma M. (2023). A phase 2 study (DisTinGuish) of DKN-01 in combination with tislelizumab + chemotherapy as first-line (1L) therapy in patients with advanced gastric or GEJ adenocarcinoma (GEA). J. Clin. Oncol..

[75] Arend R., Dholakia J., Castro C., Matulonis U., Hamilton E., Jackson C.G., LyBarger K., Goodman H.M., Duska L.R., Mahdi H. (2023). DKK1 is a predictive biomarker for response to DKN-01: Results of a phase 2 basket study in women with recurrent endometrial carcinoma. Gynecol. Oncol..

[76] Soerensen P.G., Andersson T., Buhl U., Moelvadgaard T., Jensen P.B., Brunner N., Nielsen D. (2014). Phase I dose-escalating study to evaluate the safety, tolerability, and pharmacokinetic and pharmacodynamic profiles of Foxy-5 in patients with metastatic breast, colorectal, or prostate cancer. J. Clin. Oncol..

[77] Choi M.Y., Widhopf G.F., Ghia E.M., Kidwell R.L., Hasan M.K., Yu J., Rassenti L.Z., Chen L., Chen Y., Pittman E. (2018). Phase I Trial: Cirmtuzumab Inhibits ROR1 Signaling and Stemness Signatures in Patients with Chronic Lymphocytic Leukemia. Cell Stem Cell.

[78] Shatsky R.A., Batra-Sharma H., Helsten T., Schwab R.B., Pittman E.I., Pu M., Weihe E., Ghia E.M., Rassenti L.Z., Molinolo A. (2024). A phase 1b study of zilovertamab in combination with paclitaxel for locally advanced/unresectable or metastatic HER2-negative breast cancer. Breast Cancer Res..

[79] Kipps T.J. (2021). Mining the microenvironment for therapeutic targets in chronic lymphocytic leukemia. Cancer J..

[80] Thompson P.A., Huang C., Yang J., Wertheim B.C., Roe D., Zhang X., Ding J., Chalasani P., Preece C., Martinez J. (2021). Sulindac, a Nonselective NSAID, Reduces Breast Density in Postmenopausal Women with Breast Cancer Treated with Aromatase Inhibitors. Clin. Cancer Res..

[81] Limburg P.J., Mandrekar S.J., Aubry M.C., Ziegler K.L., Zhang J., Yi J.E., Henry M., Tazelaar H.D., Lam S., McWilliams A. (2013). Cancer Prevention Network. Randomized phase II trial of sulindac for lung cancer chemoprevention. Lung Cancer.

[82] Inglis D.J., Licari J., Georgiou K.R., Wittwer N.L., Hamilton R.W., Beaumont D.M., Scherer M.A., Lavranos T.C. (2018). Characterization of BNC101 a human specific monoclonal antibody targeting the GPCR LGR5: First-in-human evidence of target engagement. Cancer Res..

[83] Cranmer L.D., Abdul Razak A.R., Ratan R., Choy E., George S., Liebner D.A., Stenehjem D.D., Gounder M.M. (2022). Results of a phase I dose escalation and expansion study of tegavivint (BC2059), a first-in-class TBL1 inhibitor for patients with progressive, unresectable desmoid tumor. J. Clin. Oncol..

[84] Plummer R., Dua D., Cresti N., Drew Y., Stephens P., Foegh M., Knudsen S., Sachdev P., Mistry B.M., Dixit V. (2020). First-in-human study of the PARP/tankyrase inhibitor E7449 in patients with advanced solid tumors and evaluation of a novel drug-response predictor. Br. J. Cancer.

[85] Scuoppo C., Ghamsari L., Gallagher E., Leong S., Koester M., Ramirez R., Gonzales J., Merutka G., Kappel B., Vainstein-Haras A. (2023). Immunotherapeutic potential of ST316, a peptide antagonist of β-catenin. Cancer Res..

[86] Ghamsari L., Gallagher E., Darvishi E., Leong S., Koester M., Ramirez R., Gonzales J., Merutka G., Kappel B., Rotolo J.A. (2022). β-catenin antagonist peptide, ST316, attenuates Wnt-dependent oncogenic activity. Cancer Res..

[87] Osada T., Chen M., Yang X.Y., Spasojevic I., Vandeusen J.B., Hsu D., Clary B.M., Clay T.M., Chen W., Morse M.A. (2011). Antihelminth compound niclosamide downregulates Wnt signaling and elicits antitumor responses in tumors with activating APC mutations. Cancer Res..

[88] Burock S., Daum S., Keilholz U., Neumann K., Walther W., Stein U. (2018). Phase II trial to investigate the safety and efficacy of orally applied niclosamide in patients with metachronous or sychronous metastases of a colorectal cancer progressing after therapy: The NIKOLO trial. BMC Cancer.

[89] Schweizer M.T., Haugk K., McKiernan J.S., Gulati R., Cheng H.H., Maes J.L., Dumpit R.F., Nelson P.S., Montgomery B., McCune J.S. (2018). A phase I study of niclosamide in combination with enzalutamide in men with castration-resistant prostate cancer. PLoS ONE.

[90] Mehnert J.M., Silk A.W., Lee J.H., Dudek L., Jeong B.S., Li J., Schenkel J.M., Sadimin E., Kane M., Lin H. (2018). A phase II trial of riluzole, an antagonist of metabotropic glutamate receptor 1 (GRM1) signaling, in patients with advanced melanoma. Pigment Cell Melanoma Res..

[91] Holtzhausen A., Zhao F., Evans K.S., Tsutsui M., Orabona C., Tyler D.S., Hanks B.A. (2015). Melanoma-Derived Wnt5a Promotes Local Dendritic-Cell Expression of IDO and Immunotolerance: Opportunities for Pharmacologic Enhancement of Immunotherapy. Cancer Immunol. Res..

[92] You L., He B., Xu Z., Uematsu K., Mazieres J., Fujii N., Mikami I., Reguart N., McIntosh J.K., Kashani-Sabet M. (2004). An anti-Wnt-2 monoclonal antibody induces apoptosis in malignant melanoma cells and inhibits tumor growth. Cancer Res..

[93] Fujii N., You L., Xu Z., Uematsu K., Shan J., He B., Mikami I., Edmondson L.R., Neale G., Zheng J. (2007). An antagonist of dishevelled protein-protein interaction suppresses beta-catenin-dependent tumor cell growth. Cancer Res..

[94] Mariotti L., Pollock K., Guettler S. (2017). Regulation of Wnt/β-catenin signalling by tankyrase-dependent poly (ADP-ribosyl)ation and scaffolding. Br. J. Pharmacol..

[95] Ferri M., Liscio P., Carotti A., Asciutti S., Sardella R., Macchiarulo A., Camaioni E. (2017). Targeting Wnt-driven cancers: Discovery of novel tankyrase inhibitors. Eur. J. Med. Chem..

[96] Damale M.G., Pathan S.K., Shinde D.B., Patil R.H., Arote R.B., Sangshetti J.N. (2020). Insights of tankyrases: A novel target for drug discovery. Eur. J. Med. Chem..

[97] Antony F., Kang X., Pundkar C., Wang C., Mishra A., Chen P., Babu R.J., Suryawanshi A. (2023). Targeting β-catenin using XAV939 nanoparticle promotes immunogenic cell death and suppresses conjunctival melanoma progression. Int. J. Pharm..

[98] Waaler J., Mygland L., Tveita A., Strand M.F., Solberg N.T., Olsen P.A., Aizenshtadt A., Fauskanger M., Lund K., Brinch S.A. (2020). Tankyrase inhibition sensitizes melanoma to PD-1 immune checkpoint blockade in syngeneic mouse models. Commun. Biol..

[99] Yan M., Li G., An J. (2017). Discovery of small molecule inhibitors of the Wnt/β-catenin signaling pathway by targeting β-catenin/Tcf4 interactions. Exp. Biol. Med..

[100] Sinnberg T., Menzel M., Ewerth D., Sauer B., Schwarz M., Schaller M., Garbe C., Schittek B. (2011). β-Catenin signaling increases during melanoma progression and promotes tumor cell survival and chemoresistance. PLoS ONE.

[101] Sinnberg T., Levesque M.P., Krochmann J., Cheng P.F., Ikenberg K., Meraz-Torres F., Niessner H., Garbe C., Busch C. (2018). Wnt-signaling enhances neural crest migration of melanoma cells and induces an invasive phenotype. Mol. Cancer.

[102] Talar B., Gajos-Michniewicz A., Talar M., Chouaib S., Czyz M. (2016). Pentoxifylline inhibits wnt signalling in β-Catenin^high^ patient-derived melanoma cell populations. PLoS ONE.

[103] Biechele T.L., Camp N.D., Fass D.M., Kulikauskas R.M., Robin N.C., White B.D., Taraska C.M., Moore E.C., Muster J., Karmacharya R. (2010). Chemical-genetic screen identifies riluzole as an enhancer of Wnt/β-catenin signaling in melanoma. Chem. Biol..

[104] Atkinson J.M., Rank K.B., Zeng Y., Capen A., Yadav V., Manro J.R., Engler T.A., Chedid M. (2015). Activating the Wnt/β-Catenin Pathway for the Treatment of Melanoma-Application of LY2090314, a Novel Selective Inhibitor of Glycogen Synthase Kinase-3. PLoS ONE.

[105] De Jaeghere E.A., Tuyaerts S., Van Nuffel A.M.T., Belmans A., Bogaerts K., Baiden-Amissah R., Lippens L., Vuylsteke P., Henry S., Trinh X.B. (2023). Pembrolizumab, radiotherapy, and an immunomodulatory five-drug cocktail in pretreated patients with persistent, recurrent, or metastatic cervical or endometrial carcinoma: Results of the phase II PRIMMO study. Cancer Immunol. Immunother..

[106] Tuyaerts S., Van Nuffel A.M.T., Naert E., Van Dam P.A., Vuylsteke P., De Caluwe A., Aspeslagh S., Dirix P., Lippens L., De Jaeghere E. (2019). PRIMMO study protocol: A phase II study combining PD-1 blockade, radiation and immunomodulation to tackle cervical and uterine cancer. BMC Cancer.

[107] Gbolahan O.B., O’Neil B.H., McRee A.J., Sanoff H.K., Fallon J.K., Smith P.C., Ivanova A., Moore D.T., Dumond J., Asher G.N. (2022). A phase I evaluation of the effect of curcumin on dose-limiting toxicity and pharmacokinetics of irinotecan in participants with solid tumors. Clin. Transl. Sci..

[108] Lev-Ari S., Strier L., Kazanov D., Madar-Shapiro L., Dvory-Sobol H., Pinchuk I., Marian B., Lichtenberg D., Arber N. (2005). Celecoxib and curcumin synergistically inhibit the growth of colorectal cancer cells. Clin. Cancer Res..

[109] Gunther J.R., Chadha A.S., Guha S., Raju G.S., Maru D.M., Munsell M.F., Jiang Y., Yang P., Felix E., Clemons M. (2022). A phase II randomized double blinded trial evaluating the efficacy of curcumin with pre-operative chemoradiation for rectal cancer. J. Gastrointest. Oncol..

[110] Srivastava N.S., Srivastava R.A.K. (2019). Curcumin and quercetin synergistically inhibit cancer cell proliferation in multiple cancer cells and modulate Wnt/β-catenin signaling and apoptotic pathways in A375 cells. Phytomedicine.

[111] Prasad C.P., Rath G., Mathur S., Bhatnagar D., Ralhan R. (2009). Potent growth suppressive activity of curcumin in human breast cancer cells: Modulation of Wnt/beta-catenin signaling. Chem. Biol. Interact..

[112] Li X., Wang X., Xie C., Zhu J., Meng Y., Chen Y., Zhao Y. (2018). Sonic hedgehog and Wnt/β-catenin pathways mediate curcumin inhibition of breast cancer stem cells. Anti-Cancer Drugs.

[113] Wang J.Y., Wang X., Wang X.J., Zheng B.Z., Wang Y., Wang X. (2018). Curcumin inhibits the growth via Wnt/beta-catenin pathway in non-small-cell lung cancer cells. Eur. Rev. Med. Pharmacol. Sci..

[114] He M., Li Y., Zhang L., Li L., Shen Y., Lin L., Zheng W., Chen L., Bian X., Ng H.K. (2014). Curcumin suppresses cell proliferation through inhibition of the Wnt/beta-catenin signaling pathway in medulloblastoma. Oncol. Rep..

[115] Shi J., Wang Y., Jia Z., Gao Y., Zhao C., Yao Y. (2017). Curcumin inhibits bladder cancer progression via regulation of β-catenin expression. Tumour Biol..

[116] Zheng R., Deng Q., Liu Y., Zhao P. (2017). Curcumin inhibits gastric carcinoma cell growth and induces apoptosis by suppressing the Wnt/β-Catenin signaling pathway. Med. Sci. Monit..

[117] Dou H., Shen R., Tao J., Huang L., Shi H., Chen H. (2017). Curcumin suppresses the colon cancer proliferation by inhibiting Wnt/beta-catenin pathways via miR-130a. Front. Pharmacol..

[118] Marjaneh R.M., Rahmani F., Hassanian S.M., Rezaei N., Hashemzehi M., Bahrami A. (2018). Phytosomal curcumin inhibits tumor growth in colitis-associated colorectal cancer. J. Cell Physiol..

[119] Yen H.Y., Tsao C.W., Lin Y.W., Kuo C.C., Tsao C.H., Liu C.Y. (2019). Regulation of carcinogenesis and modulation through Wnt/β-catenin signaling by curcumin in an ovarian cancer cell line. Sci. Rep..

[120] Pintova S., Dharmupari S., Moshier E., Zubizarreta N., Ang C., Holcombe R.F. (2019). Genistein combined with FOLFOX or FOLFOX-Bevacizumab for the treatment of metastatic colorectal cancer: Phase I/II pilot study. Cancer Chemother. Pharmacol..

[121] Howells L.M., Iwuji C.O.O., Irving G.R.B., Barber S., Walter H., Sidat Z., Griffin-Teall N., Singh R., Foreman N., Patel S.R. (2019). Curcumin Combined with FOLFOX Chemotherapy Is Safe and Tolerable in Patients with Metastatic Colorectal Cancer in a Randomized Phase IIa Trial. J. Nutr..

[122] Nguyen A.V., Martinez M., Stamos M.J., Moyer M.P., Planutis K., Hope C., Holcombe R.F. (2009). Results of a phase I pilot clinical trial examining the effect of plant-derived resveratrol and grape powder on Wnt pathway target gene expression in colonic mucosa and colon cancer. Cancer Manag. Res..

[123] Howells L.M., Berry D.P., Elliott P.J., Jacobson E.W., Hoffmann E., Hegarty B., Brown K., Steward W.P., Gescher A.J. (2011). Phase I randomized, double-blind pilot study of micronized resveratrol (SRT501) in patients with hepatic metastases--safety, pharmacokinetics, and pharmacodynamics. Cancer Prev. Res..

[124] Zhu J., Jiang Y., Yang X., Wang S., Xie C., Li X., Li Y., Chen Y., Wang X., Meng Y. (2017). Wnt/β-catenin pathway mediates (−)-Epigallocatechin-3-gallate (EGCG) inhibition of lung cancer stem cells. Biochem. Biophys. Res. Commun..

[125] Yang C., Du W., Yang D. (2016). Inhibition of green tea polyphenol ((−)-epigallocatechin-3-gallate) on the proliferation of gastric cancer cells by suppressing canonical wnt/β-catenin signalling pathway. Int. J. Food Sci. Nutr..

[126] Chen Y., Wang X.Q., Zhang Q., Zhu J.Y., Li Y., Xie C.F., Li X.T., Wu J.S., Geng S.S., Zhong C.Y. (2017). (−)-Epigallocatechin-3-Gallate Inhibits Colorectal Cancer Stem Cells by Suppressing Wnt/β-Catenin Pathway. Nutrients.

[127] Singh T., Katiyar S.K. (2013). Green tea polyphenol, (−)-epigallocatechin-3-gallate, induces toxicity in human skin cancer cells by targeting β-catenin signaling. Toxicol. Appl. Pharmacol..

[128] Kim J., Zhang X., Rieger-Christ K.M., Summerhayes I.C., Wazer D.E., Paulson K.E., Yee A.S. (2006). Suppression of Wnt signaling by the green tea compound (−)-epigallocatechin 3-gallate (EGCG) in invasive breast cancer cells: Requirement of the transcriptional repressor HBP1. J. Biol. Chem..

[129] Xu M., Wang S., Song Y.U., Yao J., Huang K., Zhu X. (2016). Apigenin suppresses colorectal cancer cell proliferation, migration and invasion via inhibition of the Wnt/β-catenin signaling pathway. Oncol. Lett..

[130] Lin C.M., Chen H.H., Lin C.A., Wu H.C., Sheu J.J., Chen H.J. (2017). Apigenin-induced lysosomal degradation of β-catenin in Wnt/β-catenin signaling. Sci. Rep..

[131] Pan F.F., Zheng Y.B., Shi C.J., Zhang F.W., Zhang J.F., Fu W.M. (2021). H19-Wnt/β-catenin regulatory axis mediates the suppressive effects of apigenin on tumor growth in hepatocellular carcinoma. Europ. J. Pharmacol..

[132] Jia Y., Chen L., Guo S., Li Y. (2019). Baicalin induced colon cancer cells apoptosis through miR-217/DKK1-mediated inhibition of Wnt signaling pathway. Mol. Biol. Rep..

[133] Örenlili Yaylagül E., Ülger C. (2020). The effect of baicalein on Wnt/β-catenin pathway and miR-25 expression in Saos-2 osteosarcoma cell line. Turk. J. Med. Sci..

[134] Xia X., Xia J., Yang H., Li Y., Liu S., Cao Y., Tang L., Yu X. (2019). Baicalein blocked cervical carcinoma cell proliferation by targeting CCND1 via Wnt/β-catenin signaling pathway. Artif. Cells Nanomed. Biotechnol..

[135] Liu X., Liu S., Chen J., He L., Meng X., Liu S. (2016). Baicalein suppresses the proliferation of acute T-lymphoblastic leukemia Jurkat cells by inhibiting the Wnt/β-catenin signaling. Ann. Hematol..

[136] Zhou T., Zhang A., Kuang G., Gong X., Jiang R., Lin D., Li J., Li H., Zhang X., Wan J. (2017). Baicalin inhibits the metastasis of highly aggressive breast cancer cells by reversing epithelial-to-mesenchymal transition by targeting β-catenin signaling. Oncol. Rep..

[137] Gwak J., Oh J., Cho M., Bae S.K., Song I.S., Liu K.H., Jeong Y., Kim D.E., Chung Y.H., Oh S. (2011). Galangin suppresses the proliferation of β-catenin response transcription-positive cancer cells by promoting adenomatous polyposis coli/Axin/glycogen synthase kinase-3β-independent β-catenin degradation. Molecul. Pharm..

[138] Ren K., Zhang W., Wu G., Ren J., Lu H., Li Z., Han X. (2016). Synergistic anti-cancer effects of galangin and berberine through apoptosis induction and proliferation inhibition in oesophageal carcinoma cells. Biomed. Pharmacother..

[139] Lu W., Lin C., King T.D., Chen H., Reynolds R.C., Li Y. (2012). Silibinin inhibits Wnt/β-catenin signaling by suppressing Wnt co-receptor LRP6 expression in human prostate and breast cancer cells. Cell Signal..

[140] Sameri S., Saidijam M., Bahreini F., Najafi R. (2021). Cancer chemopreventive activities of silibinin on colorectal cancer through regulation of E-Cadherin/β-Catenin pathway. Nutr. Cancer.

[141] Park S., Choi J. (2010). Inhibition of beta-catenin/Tcf signaling by flavonoids. J. Cell Biochem..

[142] Pu Y., Han Y., Ouyang Y., Li H., Li L., Wu X., Yang L., Gao J., Zhang L., Zhou J. (2024). Kaempferol inhibits colorectal cancer metastasis through circ_0000345 mediated JMJD2C/β-catenin signalling pathway. Phytomedicine.

[143] Qin B., Liu J.W., Liu S.W., Li B.J., Ren J. (2016). Kaempferol targets estrogen-related receptor alpha and inhibits cell proliferation and invasion in retinoblastoma via Wnt/beta-catenin signaling pathway. Int. J. Clin. Exp. Med..

[144] Kim M., Kim S.H., Lim J.W., Kim H. (2019). Lycopene induces apoptosis by inhibiting nuclear translocation of β-catenin in gastric cancer cells. J. Physiol. Pharmacol..

[145] Preet R., Mohapatra P., Das D., Satapathy S.R., Choudhuri T., Wyatt M.D., Kundu C.N. (2013). Lycopene synergistically enhances quinacrine action to inhibit Wnt-TCF signaling in breast cancer cells through APC. Carcinogenesis.

[146] Zhu H., Zou X., Lin S., Hu X., Gao J. (2020). Effects of naringin on reversing cisplatin resistance and the Wnt/β-catenin pathway in human ovarian cancer SKOV3/CDDP cells. J. Int. Med. Res..

[147] Kang Q., Gong J., Wang M., Wang Q., Chen F., Cheng K.W. (2019). 6-C-(E-Phenylethenyl) Naringenin Attenuates the Stemness of Hepatocellular Carcinoma Cells by Suppressing Wnt/β-Catenin Signaling. J. Agric. Food Chem..

[148] Tong Y., Liu Y., Zheng H., Zheng L., Liu W., Wu J., Ou R., Zhang G., Li F., Hu M. (2016). Artemisinin and its derivatives can significantly inhibit lung tumorigenesis and tumor metastasis through Wnt/β-catenin signaling. Oncotarget.

[149] Park C.H., Chang J.Y., Hahm E.R., Park S., Kim H.K., Yang C.H. (2005). Quercetin, a potent inhibitor against beta-catenin/Tcf signaling in SW480 colon cancer cells. Biochem. Biophys. Res. Commun..

[150] Jung Y., Shin S.Y., Lee Y.H., Lim Y. (2017). Flavones with inhibitory effects on glycogen synthase kinase 3β. Appl. Biol. Chem..

[151] Gonçalves C.F.L., Hecht F., Cazarin J., Fortunato R.S., Vaisman M., Carvalho D.P., Ferreira A.C.F. (2021). The flavonoid quercetin reduces cell migration and increases NIS and E-cadherin mRNA in the human thyroid cancer cell line BCPAP. Mol. Cell Endocrinol..

[152] Syed D.N., Afaq F., Maddodi N., Johnson J.J., Sarfaraz S., Ahmad A., Setaluri V., Mukhtar H. (2011). Inhibition of human melanoma cell growth by the dietary flavonoid fisetin is associated with disruption of Wnt/β-catenin signaling and decreased Mitf levels. J. Investig. Dermatol..

[153] Suh Y., Afaq F., Johnson J.J., Mukhtar H. (2009). A plant flavonoid fisetin induces apoptosis in colon cancer cells by inhibition of COX2 and Wnt/EGFR/NF-kappaB-signaling pathways. Carcinogenesis.

[154] Hu J., Guo X., Yang L. (2018). Morin inhibits proliferation and self-renewal of CD133^+^ melanoma cells by upregulating miR-216a. J. Pharmacol. Sci..

[155] Du M., Shen P., Tan R., Wu D., Tu S. (2021). Aloe-emodin inhibits the proliferation, migration, and invasion of melanoma cells via inactivation of the Wnt/beta-catenin signaling pathway. Ann. Transl. Med..

[156] Hussain T., Alafnan A., Almazni I.A., Helmi N., Moin A., Baeissa H.M., Awadelkareem A.M., Elkhalifa A.O., Bakhsh T., Alzahrani A. (2024). Aloe-emodin exhibits growth-suppressive effects on androgen-independent human prostate cancer DU145 cells via inhibiting the Wnt/β-catenin signaling pathway: An in vitro and in silico study. Front. Pharmacol..

[157] Tarapore R.S., Siddiqui I.A., Saleem M., Adhami V.M., Spiegelman V.S., Mukhtar H. (2010). Specific targeting of Wnt/β-catenin signaling in human melanoma cells by a dietary triterpene lupeol. Carcinogenesis.

[158] Huang L., Zhao Z., Li C. (2023). Lupeol Inhibits Stemness in Colon Cancer-Stem Like Cells and Promotes Chemosensitivity via Degrading β-Catenin. J. Biol. Regul. Homeost. Agents.

[159] Zhang L., Tu Y., He W., Peng Y., Qiu Z. (2015). A novel mechanism of hepatocellular carcinoma cell apoptosis induced by lupeol via brain-derived neurotrophic factor inhibition and glycogen synthase kinase 3 beta reactivation. Eur. J. Pharmacol..

[160] Maurya S.K., Fatma H., Maurya A.K., Mishra N., Siddique H.R. (2022). Role of lupeol in chemosensitizing therapy-resistant prostate cancer cells by targeting MYC, β-catenin and c-FLIP: In silico and in vitro studies. In Silico Pharmacol..

[161] Zhang L., Chen J., Chen Y., Zou D., Pu Y., Wei M., Huang Y., Li Y., Huang Q., Chen J. (2023). Alantolactone inhibits melanoma cell culture viability and migration and promotes apoptosis by inhibiting Wnt/β-catenin signaling. Anticancer Agents Med. Chem..

[162] Yang C., Zhang L., Huang H., Yuan X., Zhang P., Ye C., Wei M., Huang Y., Luo X., Luo J. (2020). Alantolactone inhibits proliferation, metastasis and promotes apoptosis of human osteosarcoma cells by suppressing Wnt/β-catenin and MAPKs signaling pathways. Genes Dis..

[163] Shabna A., Antony J., Vijayakurup V., Saikia M., Liju V.B., Retnakumari A.P., Amrutha N.A., Alex V.V., Swetha M., Aiswarya S.U. (2022). Pharmacological attenuation of melanoma by tryptanthrin pertains to the suppression of MITF-M through MEK/ERK signaling axis. Cell. Mol. Life Sci..

[164] Wang G., Wang Y., Yao L., Gu W., Zhao S., Shen Z., Lin Z., Liu W., Yan T. (2022). Pharmacological activity of quercetin: An updated review. Evid. Based Complement Alternat. Med..

[165] Alizadeh S.R., Ebrahimzadeh M.A. (2022). Quercetin derivatives: Drug design, development, and biological activities, a review. Eur. J. Med. Chem..

[166] Zhu X., Ma P., Peng D., Wang Y., Wang D., Chen X., Zhang X., Song Y. (2016). Quercetin suppresses lung cancer growth by targeting Aurora B kinase. Cancer Med..

[167] Mirzaei A., Deyhimfar R., Azodian Ghajar H., Mashhadi R., Noori M., Dialameh H., Aghsaeifard Z., Aghamir S.M.K. (2023). Quercetin can be a more reliable treatment for metastatic prostate cancer than the localized disease: An in vitro study. J. Cell. Mol. Med..

[168] Hisaka T., Sakai H., Sato T., Goto Y., Nomura Y., Fukutomi S., Fujita F., Mizobe T., Nakashima O., Tanigawa M. (2020). Quercetin suppresses proliferation of liver cancer cell lines in vitro. Anticancer Res..

[169] Wang R., Yang L., Li S., Ye D., Yang L., Liu Q., Zhao Z., Cai Q., Tan J., Li X. (2018). Quercetin inhibits breast cancer stem cells via downregulation of aldehyde dehydrogenase 1A1 (ALDH1A1), chemokine receptor type 4 (CXCR4), mucin 1 (MUC1), and epithelial cell adhesion molecule (EpCAM). Med. Sci. Monit..

[170] Tezerji S., Nazari Robati F., Abdolazimi H., Fallah A., Talaei B. (2022). Quercetin’s effects on colon cancer cells apoptosis and proliferation in a rat model of disease. Clin. Nutr. ESPEN.

[171] He C., Lu X., Li J., Shen K., Bai Y., Li Y., Luan H., Tuo S. (2021). The effect of quercetin on cervical cancer cells as determined by inducing tumor endoplasmic reticulum stress and apoptosis and its mechanism of action. Am. J. Transl. Res..

[172] Asgharian P., Tazekand A.P., Hosseini K., Forouhandeh H., Ghasemnejad T., Ranjbar M., Hasan M., Kumar M., Beirami S.M., Tarhriz V. (2022). Potential mechanisms of quercetin in cancer prevention: Focus on cellular and molecular targets. Cancer Cell Int..

[173] Sultan A.S., Khalil M.I.M., Sami B.M., Alkhuriji A.F., Sadek O. (2017). Quercetin induces apoptosis in triple-negative breast cancer cells via inhibiting fatty acid synthase and beta-catenin. Int. J. Clin. Exp. Pathol..

[174] Li H., Li M., Fu J., Ao H., Wang W., Wang X. (2021). Enhancement of oral bioavailability of quercetin by metabolic inhibitory nanosuspensions compared to conventional nanosuspensions. Drug Deliv..

[175] Pal H.C., Diamond A.C., Strickland L.R., Kappes J.C., Katiyar S.K., Elmets C.A., Athar M., Afaq F. (2016). Fisetin, a dietary flavonoid, augments the anti-invasive and anti-metastatic potential of sorafenib in melanoma. Oncotarget.

[176] Kumar R.M., Kumar H., Bhatt T., Jain R., Panchal K., Chaurasiya A., Jain V. (2023). Fisetin in cancer: Attributes, developmental aspects, and nanotherapeutics. Pharmaceuticals.

[177] Zhou C., Huang Y., Nie S., Zhou S., Gao X., Chen G. (2023). Biological effects and mechanisms of fisetin in cancer: A promising anti-cancer agent. Eur. J. Med. Res..

[178] Sabarwal A., van Rooyen J.C., Caburet J., Avgenikos M., Dheeraj A., Ali M., Mishra D., de Meester J.S.B., Stander S., van Otterlo W.A.L. (2022). A novel 4′-brominated derivative of fisetin induces cell cycle arrest and apoptosis and inhibits EGFR/ERK1/2/STAT3 pathways in non-small-cell lung cancer without any adverse effects in mice. FASEB J..

[179] Tabasum S., Singh R.P. (2019). Fisetin suppresses migration, invasion and stem-cell-like phenotype of human non-small cell lung carcinoma cells via attenuation of epithelial to mesenchymal transition. Chem. Biol. Interact..

[180] Klimaszewska-Wisniewska A., Halas-Wisniewska M., Grzanka A., Grzanka D. (2018). Evaluation of anti-metastatic potential of the combination of fisetin with paclitaxel on A549 non-small cell lung cancer cells. Int. J. Mol. Sci..

[181] Sun X., Ma X., Li Q., Yang Y., Xu X., Sun J., Yu M., Cao K., Yang L., Yang G. (2018). Anti-cancer effects of fisetin on mammary carcinoma cells via regulation of the PI3K/Akt/mTOR pathway: In vitro and in vivo studies. Int. J. Mol. Med..

[182] Mukhtar E., Adhami V.M., Siddiqui I.A., Verma A.K., Mukhtar H. (2016). Fisetin enhances chemotherapeutic effect of cabazitaxel against human prostate cancer cells. Mol. Cancer Ther..

[183] Jeng L.B., Kumar Velmurugan B., Chen M.C., Hsu H.H., Ho T.J., Day C.H., Huang C.Y. (2018). Fisetin mediated apoptotic cell death in parental and Oxaliplatin/irinotecan resistant colorectal cancer cells in vitro and in vivo. J. Cell. Physiol..

[184] Li J., Qu W., Cheng Y., Sun Y., Jiang Y., Zou T., Wang Z., Xu Y., Zhao H. (2014). The inhibitory effect of intravesical fisetin against bladder cancer by induction of p53 and down-regulation of NF-kappa B pathways in a rat bladder carcinogenesis model. Basic Clin. Pharmacol. Toxicol..

[185] Si Y., Liu J., Shen H., Zhang C., Wu Y., Huang Y., Liu T. (2019). Fisetin decreases TET 1 activity and CCNY/CDK 16 promoter 5hmC levels to inhibit the proliferation and invasion of renal cancer stem cell. J. Cell. Mol. Med..

[186] Ferreira de Oliveira J.M.P., Pacheco A.R., Coutinho L., Oliveira H., Pinho S., Almeida L., Santos C. (2018). Combination of etoposide and fisetin results in anti-cancer efficiency against osteosarcoma cell models. Arch. Toxicol..

[187] Jia S., Xu X., Zhou S., Chen Y., Ding G., Cao L. (2019). Fisetin in-duces autophagy in pancreatic cancer cells via endoplasmic reticulum stress-and mitochondrial stress dependent pathways. Cell Death Dis..

[188] Kim N., Kang M.J., Lee S.H., Son J.H., Lee J.E., Paik W.H., Kim Y.T. (2018). Fisetin enhances the cytotoxicity of gemcitabine by down-regulating ERK-MYC in MiaPaca-2 human pancreatic cancer cells. Anticancer Res..

[189] Liu X.F., Long H.J., Miao X.Y., Liu G.L., Yao H.L. (2017). Fisetin inhibits liver cancer growth in a mouse model: Relation to dopamine receptor. Oncol. Rep..

[190] Park B.S., Choi N.E., Lee J.H., Kang H.M., Yu S.B., Kim H.J., Kang H.K., Kim I.R. (2019). Crosstalk between fisetin-induced apoptosis and autophagy in human oral squamous cell carcinoma. J. Cancer.

[191] Yan W., Chen S., Zhao Y., Ye X. (2018). Fisetin inhibits the proliferation of gastric cancer cells and induces apoptosis through sup-pression of ERK 1/2 activation. Oncol. Lett..

[192] Sak K., Kasemaa K., Everaus H. (2016). Potentiation of luteolin cytotoxicity by flavonols fisetin and quercetin in human chronic lympho-cytic leukemia cell lines. Food Funct..

[193] Xiao X., Zou J., Fang Y., Meng Y., Xiao C., Fu J., Yao Y. (2018). Fisetin and polymeric micelles encapsulating fisetin exhibit potent cytotoxic effects towards ovarian cancer cells. BMC Complement Altern. Med..

[194] Lin M.T., Lin C.L., Lin T.Y., Cheng C.W., Yang S.F., Lin C.L., Wu C.C., Hsieh Y.H., Tsai J.P. (2016). Synergistic effect of fisetin combined with sorafenib in human cervical cancer HeLa cells through activation of death recep-tor-5 mediated caspase-8/caspase-3 and the mitochondria-depen-dent apoptotic pathway. Tumour Biol..

[195] Molagoda I.M.N., Karunarathne W.A.H.M., Park S.R., Choi Y.H., Park E.K., Jin C.Y., Yu H., Jo W.S., Lee K.T., Kim G.Y. (2020). GSK-3β-targeting fisetin promotes melanogenesis in B16F10 melanoma cells and zebrafish larvae through β-Catenin activation. Int. J. Mol. Sci..

[196] Balaga V.K.R., Pradhan A., Thapa R., Patel N., Mishra R., Singla N. (2023). Morin: A comprehensive review on its versatile biological activity and associated therapeutic potential in treating cancers. Pharmacol. Res. Mod. Chin. Med..

[197] Maharjan S., Lee M.G., Kim S.Y., Lee K.S., Nam K.S. (2023). Morin sensitizes MDA-MB-231 triple-negative breast cancer cells to doxorubicin cytotoxicity by suppressing FOXM1 and attenuating EGFR/STAT3 signaling pathways. Pharmaceuticals.

[198] Xu M., Zhang Y. (2019). Morin inhibits ovarian cancer growth through the inhibition of nf-κb signaling pathway. Anticancer Agents Med. Chem..

[199] Gor R., Saha L., Agarwal S., Karri U., Sohani A., Madhavan T., Pachaiappan R., Ramalingam S. (2022). Morin inhibits colon cancer stem cells by inhibiting PUM1 expression in vitro. Med. Oncol..

[200] Pal Singh M., Pal Khaket T., Bajpai V.K., Alfarraj S., Kim S.G., Chen L., Huh Y.S., Han Y.K., Kang S.C. (2020). Morin hydrate sensitizes hepatoma cells and xenograft tumor towards cisplatin by downregulating PARP-1-HMGB1 mediated autophagy. Int. J. Mol. Sci..

[201] Yao D., Cui H., Zhou S., Guo L. (2017). Morin inhibited lung cancer cells viability, growth, and migration by suppressing miR-135b and inducing its target CCNG2. Tumour Biol..

[202] Pereira W.L., de Oliveira T.T., Kanashiro M.M., Filardi M.A., da Costa M.R., da Costa L.M. (2021). Morin exhibits leukemic cellular apoptosis through caspase pathway. Nat. Prod. Res..

[203] Lee Y.J., Kim W.I., Kim S.Y., Cho S.W., Nam H.S., Lee S.H., Cho M.K. (2019). Flavonoid morin inhibits proliferation and induces apoptosis of melanoma cells by regulating reactive oxygen species, Sp1 and Mcl-1. Arch. Pharmacal. Res..

[204] Choi Y.A., Yoon Y.H., Choi K., Kwon M., Goo S.H., Cha J.S., Choi M.K., Lee H.S., Song I.S. (2015). Enhanced oral bioavailability of morin administered in mixed micelle formulation with PluronicF127 and Tween80 in rats. Biol. Pharm. Bull..

[205] Dong X., Zeng Y., Liu Y., You L., Yin X., Fu J., Ni J. (2020). Aloe-emodin: A review of its pharmacology, toxicity, and pharmacokinetics. Phytother. Res..

[206] Lin J.G., Chen G.W., Li T.M., Chouh S.T., Tan T.W., Chung J.G. (2006). Aloe-emodin induces apoptosis in T24 human bladder cancer cells through the p53 dependent apoptotic pathway. Urol. J..

[207] Gao R., Wu X., Huang Z., Wang B., Li F., Xu H., Ran L. (2019). Anti-tumor effect of aloe-emodin on cervical cancer cells was associated with human papillomavirus E6/E7 and glucose metabolism. Onco Targets Ther..

[208] Jiang D., Ding S., Mao Z., You L., Ruan Y. (2021). Integrated analysis of potential pathways by which aloe-emodin induces the apoptosis of colon cancer cells. Cancer Cell Int..

[209] Lin H.D., Li K.T., Duan Q.Q., Chen Q., Tian S., Chu E.S.M., Bai D.Q. (2017). The effect of aloe-emodin-induced photodynamic activity on the apoptosis of human gastric cancer cells: A pilot study. Oncol. Lett..

[210] Tabolacci C., De Vita D., Facchiano A., Bozzuto G., Beninati S., Failla C.M., Di Martile M., Lintas C., Mischiati C., Stringaro A. (2023). Phytochemicals as Immunomodulatory Agents in Melanoma. Int. J. Mol. Sci..

[211] Shen F., Ge C., Yuan P. (2020). Aloe-emodin induces autophagy and apoptotic cell death in non-small cell lung cancer cells via Akt/mTOR and MAPK signaling. Eur. J. Pharmacol..

[212] Zhu M., He Q., Wang Y., Duan L., Rong K., Wu Y., Ding Y., Mi Y., Ge X., Yang X. (2023). Exploring the mechanism of aloe-emodin in the treatment of liver cancer through network pharmacology and cell experiments. Front. Pharmacol..

[213] Chen S., Guan X., Xie L., Liu C., Li C., He M., Hu J., Fan H., Li Q., Xie L. (2023). Aloe-emodin targets multiple signaling pathways by blocking ubiquitin-mediated degradation of DUSP1 in nasopharyngeal carcinoma cells. Phytother. Res..

[214] Li Q., Wen J., Yu K., Shu Y., He W., Chu H., Zhang B., Ge C. (2018). Aloe-emodin induces apoptosis in human oral squamous cell carcinoma SCC15 cells. BMC Complement Altern. Med..

[215] He T.P., Yan W.H., Mo L.E., Liang N.C. (2008). Inhibitory effect of aloe-emodin on metastasis potential in ho-8910PM cell line. J. Asian Nat. Prod. Res..

[216] Liu K., Park C., Li S., Lee K.W., Liu H., He L., Soung N.K., Ahn J.S., Bode A.M., Dong Z. (2012). Aloe-emodin suppresses prostate cancer by targeting the mTOR complex 2. Carcinogenesis.

[217] Chen Y.Y., Chiang S.Y., Lin J.G., Yang J.S., Ma Y.S., Liao C.L., Lai T.Y., Tang N.Y., Chung J.G. (2010). Emodin, aloe-emodin and rhein induced DNA damage and inhibited DNA repair gene expression in SCC-4 human tongue cancer cells. Anticancer Res..

[218] Shi F., Chen L., Wang Y., Liu J., Adu-Frimpong M., Ji H., Toreniyazov E., Wang Q., Yu J., Xu X. (2022). Enhancement of oral bioavailability and anti-hyperuricemic activity of aloe emodin via novel Soluplus^®^-glycyrrhizic acid mixed micelle system. Drug Deliv. Transl. Res..

[219] Kotha R.R., Luthria D.L. (2019). Curcumin: Biological, pharmaceutical, nutraceutical, and analytical aspects. Molecules.

[220] Hosseini-Zare M.S., Sarhadi M., Zarei M., Thilagavathi R., Selvam C. (2021). Synergistic effects of curcumin and its analogs with other bioactive compounds: A comprehensive review. Eur. J. Med. Chem..

[221] Zhao S., Pi C., Ye Y., Zhao L., Wei Y. (2019). Recent advances of analogues of curcumin for treatment of cancer. Eur. J. Med. Chem..

[222] Zang W.B., Wei H.L., Zhang W.W., Ma W., Li J., Yao Y. (2024). Curcumin hybrid molecules for the treatment of Alzheimer’s disease: Structure and pharmacological activities. Eur. J. Med. Chem..

[223] Hao J., Dai X., Gao J., Li Y., Hou Z., Chang Z., Wang Y. (2021). Curcumin suppresses colorectal tumorigenesis via the Wnt/β-catenin signaling pathway by downregulating Axin2. Oncol. Lett..

[224] Falah R.R., Talib W.H., Shbailat S.J. (2017). Combination of metformin and curcumin targets breast cancer in mice by angiogenesis inhibition, immune system modulation and induction of p53 independent apoptosis. Ther. Adv. Med. Oncol..

[225] Shao C., Wu J., Han S., Liu Y., Su Z., Zhu H.L., Liu H.K., Qian Y. (2022). Biotinylated curcumin as a novel chemosensitizer enhances naphthalimide-induced autophagic cell death in breast cancer cells. Eur. J. Med. Chem..

[226] Bilajac E., Mahmutović L., Glamočlija U., Osmanović A., Hromić-Jahjefendić A., Tambuwala M.M., Suljagić M. (2022). Curcumin decreases viability and inhibits proliferation of imatinib-sensitive and imatinib-resistant chronic myeloid leukemia cell lines. Metabolites.

[227] Li W., Sun L., Lei J., Wu Z., Ma Q., Wang Z. (2020). Curcumin inhibits pancreatic cancer cell invasion and EMT by interfering with tumor-stromal crosstalk under hypoxic conditions via the IL-6/ERK/NF-κB axis. Oncol. Rep..

[228] Rodriguez G.A., Shah A.H., Gersey Z.C., Shah S.S., Bregy A., Komotar R.J., Graham R.M. (2016). Investigating the therapeutic role and molecular biology of curcumin as a treatment for glioblastoma. Ther. Adv. Med. Oncol..

[229] Sun S., Fang H. (2021). Curcumin inhibits ovarian cancer progression by regulating circ-PLEKHM3/miR-320a/SMG1 axis. J. Ovarian Res..

[230] Kötting C., Hofmann L., Lotfi R., Engelhardt D., Laban S., Schuler P.J., Hoffmann T.K., Brunner C., Theodoraki M.N. (2021). Immune-Stimulatory Effects of Curcumin on the Tumor Microenvironment in Head and Neck Squamous Cell Carcinoma. Cancers.

[231] Shi J., Zhang X., Shi T., Li H. (2017). Antitumor effects of curcumin in human bladder cancer in vitro. Oncol Lett..

[232] Gong F., Chen D., Teng X., Ge J., Ning X., Shen Y.L., Li J., Wang S. (2017). Curcumin-Loaded Blood-Stable Polymeric Micelles for Enhancing Therapeutic Effect on Erythroleukemia. Mol. Pharm..

[233] Tang Y., Cao Y. (2022). Curcumin inhibits the growth and metastasis of melanoma via miR-222-3p/SOX10/Notch Axis. Dis. Markers.

[234] Hewlings S.J., Kalman D.S. (2017). Curcumin: A Review of its effects on human health. Foods.

[235] Anand P., Kunnumakkara A.B., Newman R.A., Aggarwal B.B. (2007). Bioavailability of curcumin: Problems and promises. Mol. Pharm..

[236] Mirzaei H., Naseri G., Rezaee R., Mohammadi M., Banikazemi Z., Mirzaei H.R., Salehi H., Peyvandi M., Pawelek J.M., Sahebkar A. (2016). Curcumin: A new candidate for melanoma therapy. Int. J. Cancer.

[237] Liu K., Zhang X., Xie L., Deng M., Chen H., Song J., Long J., Li X., Luo J. (2021). Lupeol and its derivatives as anticancer and anti-inflammatory agents: Molecular mechanisms and therapeutic efficacy. Pharmacol. Res..

[238] Zhang X., Gao Z., Chen K., Zhuo Q., Chen M., Wang J., Lai X., Wang L. (2022). Lupeol inhibits the proliferation and migration of MDA-MB-231 breast cancer cells via a novel crosstalk mechanism between autophagy and the EMT. Food Funct..

[239] Palanimuthu D., Baskaran N., Silvan S., Rajasekaran D., Manoharan S. (2012). Lupeol, a bioactive triterpene, prevents tumor formation during 7,12-dimethylbenz(a)anthracene induced oral carcinogenesis. Pathol. Oncol. Res..

[240] Bhatt M., Patel M., Adnan M., Reddy M.N. (2021). Anti-metastatic effects of lupeol via the inhibition of mapk/erk pathway in lung cancer. Anticancer Agents Med. Chem..

[241] Eldohaji L.M., Fayed B., Hamoda A.M., Ershaid M., Abdin S., Alhamidi T.B., Mohammad M.G., Omar H.A., Soliman S.S.M. (2021). Potential targeting of Hep3B liver cancer cells by lupeol isolated from Avicennia marina. Archiv. Pharmazie.

[242] Zhong J., He C., Xu F., Xu X., Liu L., Xu M., Guo Z., Wang Y., Liao J., Li Y. (2020). Lupeol inhibits osteosarcoma progression by up-regulation of HMGA2 via regulating miR-212-3p. J. Orthop. Surg. Res..

[243] Chen M.C., Hsu H.H., Chu Y.Y., Cheng S.F., Shen C.Y., Lin Y.J., Chen R.J., Viswanadha V.P., Lin Y.M., Huang C.Y. (2018). Lupeol alters ER stress-signaling pathway by downregulating ABCG2 expression to induce Oxaliplatin-resistant LoVo colorectal cancer cell apoptosis. Environ. Toxicol..

[244] Prabhu B., Balakrishnan D., Sundaresan S. (2016). Antiproliferative and anti-inflammatory properties of diindolylmethane and lupeol against N-butyl-N-(4-hydroxybutyl) nitrosamine induced bladder carcinogenesis in experimental rats. Hum. Exp. Toxicol..

[245] Bociort F., Macasoi I.G., Marcovici I., Motoc A., Grosu C., Pinzaru I., Petean C., Avram S., Dehelean C.A. (2021). Investigation of lupeol as anti-melanoma agent: An in vitro-in ovo perspective. Curr. Oncol..

[246] Saleem M., Maddodi N., Abu Zaid M., Khan N., bin Hafeez B., Asim M., Suh Y., Yun J.M., Setaluri V., Mukhtar H. (2008). Lupeol inhibits growth of highly aggressive human metastatic melanoma cells in vitro and in vivo by inducing apoptosis. Clin. Cancer Res..

[247] Sohag A.A.M., Hossain M.T., Rahaman M.A., Rahman P., Hasan M.S., Das R.C., Khan M.K., Sikder M.H., Alam M., Uddin M.J. (2022). Molecular pharmacology and therapeutic advances of the pentacyclic triterpene lupeol. Phytomedicine.

[248] Zhang J., Liang H., Yao H., Qiu Z., Chen X., Hu X., Hu J., Zheng G. (2019). The preparation, characterization of Lupeol PEGylated liposome and its functional evaluation in vitro as well as pharmacokinetics in rats. Drug Dev. Ind. Pharm..

[249] Patel S., Srivastava S., Singh M.R., Singh D. (2018). Preparation and optimization of chitosan-gelatin films for sustained delivery of lupeol for wound healing. Int. J. Biol. Macromol..

[250] Priyanka K., Kosuru R., Sharma R.P., Sahu P.L., Singh S. (2017). Assessment of pharmacokinetic parameters of lupeol in *Ficus religiosa* L. extract after oral administration of suspension and solid lipid nanoparticles to Wistar rats. Int. J. Drug Deliv. Technol..

[251] Bociort F., Crisan C.N., Dragoi R., Heghes A., Szuhanek C., Radulescu M., Berceanu-Vaduva D., Tischer A., Motoc A. (2020). Green and synthetic metallic nanoparticles—Obtaining, characterization and biological evaluation in association with lupeol. Rev. Chim..

[252] Babaei G., Gholizadeh-Ghaleh Aziz S., Rajabi Bazl M., Khadem Ansari M.H. (2021). A comprehensive review of anticancer mechanisms of action of Alantolactone. Biomed. Pharmacother..

[253] Cai Y., Gao K., Peng B., Xu Z., Peng J., Li J., Chen X., Zeng S., Hu K., Yan Y. (2021). Alantolactone: A natural plant extract as a potential therapeutic agent for cancer. Front. Pharmacol..

[254] Ren Y., Lv C., Zhang J., Zhang B., Yue B., Luo X., Yu Z., Wang H., Ren J., Wang Z. (2021). Alantolactone exhibits antiproliferative and apoptosis-promoting properties in colon cancer model via activation of the MAPK-JNK/c-Jun signaling pathway. Mol. Cell Biochem..

[255] Zhao P., Pan Z., Luo Y., Zhang L., Li X., Zhang G., Zhang Y., Cui R., Sun M., Zhang X. (2015). Alantolactone induces apoptosis and cell cycle arrest on lung squamous cancer SK-MES-1 cells. J. Biochem. Mol. Toxicol..

[256] Zhang X., Zhang H.M. (2019). Alantolactone induces gastric cancer BGC-823 cell apoptosis by regulating reactive oxygen species generation and the AKT signaling pathway. Oncol. Lett..

[257] He R., Shi X., Zhou M., Zhao Y., Pan S., Zhao C., Guo X., Wang M., Li X., Qin R. (2018). Alantolactone induces apoptosis and improves chemosensitivity of pancreatic cancer cells by impairment of autophagy-lysosome pathway via targeting TFEB. Toxicol. Appl. Pharmacol..

[258] Naderi R., Gholizadeh-Ghaleh Aziz S., Haghigi-Asl A.S. (2012). Evaluating the effect of Alantolactone on the expression of N-cadherin and Vimentin genes effective in epithelial-mesenchymal transition (EMT) in breast cancer cell line (MDA-MB-231). Ann. Med. Surg..

[259] Xu R., Peng Y., Wang M., Li X. (2019). Intestinal absorption of isoalantolactone and alantolactone, two sesquiterpene lactones from radix inulae, using Caco-2 cells. Eur. J. Drug Metab. Pharmacokinet..

[260] Zhang J., Shen L., Li X., Song W., Liu Y., Huang L. (2019). Nanoformulated codelivery of quercetin and alantolactone promotes an antitumor response through synergistic immunogenic cell death for microsatellite-stable colorectal cancer. ACS Nano.

[261] Jahng Y. (2013). Progress in the studies on tryptanthrin, an alkaloid of history. Arch. Pharm. Res..

[262] Antony J., Saikia M., Vinod V., Nath L.R., Katiki M.R., Murty M.S., Paul A., Shabna A., Chandran H., Joseph S.M. (2015). DW-F5: A novel formulation against malignant melanoma from *Wrightia tinctoria*. Sci. Rep..

[263] Kaur R., Manjal S.K., Rawal R.K., Kumar K. (2017). Recent synthetic and medicinal perspectives of tryptanthrin. Bioorg. Med. Chem..

[264] Miao S., Shi X., Zhang H., Wang S., Sun J., Hua W., Miao Q., Zhao Y., Zhang C. (2011). Proliferation-attenuating and apoptosis-inducing effects of tryptanthrin on human chronic myeloid leukemia K562 cell line in vitro. Int. J. Mol. Sci..

[265] Chan H.L., Yip H.Y., Mak N.K., Leung K.N. (2009). Modulatory effects and action mechanisms of tryptanthrin on murine myeloid leukemia cells. Cell. Mol. Immunol..

[266] Yu S.T., Chen T.M., Tseng S.Y., Chen Y.H. (2007). Tryptanthrin inhibits MDR1 and reverses doxorubicin resistance in breast cancer cells. Biochem. Biophys. Res. Commun..

[267] Li H., Fu G., Zhong W. (2023). Natural quinazolinones: From a treasure house to promising anticancer leads. Eur. J. Med. Chem..

[268] Zhu X., Zhang X., Ma G., Yan J., Wang H., Yang Q. (2011). Transport characteristics of tryptanthrin and its inhibitory effect on P-gp and MRP2 in Caco-2 cells. J. Pharm. Pharm. Sci..

[269] Shankar G.M., Alex V.V., Nisthul A.A., Bava S.V., Sundaram S., Retnakumari A.P., Chittalakkottu S., Anto R.J. (2020). Pre-clinical evidences for the efficacy of tryptanthrin as a potent suppressor of skin cancer. Cell Prolif..

[270] Fang Y.P., Lin Y.K., Su Y.H., Fang J.Y. (2011). Tryptanthrin-loaded nanoparticles for delivery into cultured human breast cancer cells, MCF7: The effects of solid lipid/liquid lipid ratios in the inner core. Chem. Pharm. Bull..

[271] Yang C., He B., Zheng Q., Wang D., Qin M., Zhang H., Dai W., Zhang Q., Meng X., Wang X. (2019). Nano-encapsulated tryptanthrin derivative for combined anticancer therapy via inhibiting indoleamine 2,3-dioxygenase and inducing immunogenic cell death. Nanomedicine.

